# Supplementary material for: BIDCell: Biologically-informed self-supervised learning for segmentation of subcellular spatial transcriptomics data
Source: Nat Commun. 2024 Jan 13;15:509. doi: 10.1038/s41467-023-44560-w (PMC10787788; doi:10.1038/s41467-023-44560-w)
Supplement: Supplementary file 1 — Supplementary Information [file 41467_2023_44560_MOESM1_ESM.pdf]

Supplementary - BIDCell

Supplementary Table 1. Summary of related methods.

Types	Method	Reference	Included	Exclusion reason
From classical approach	Dilation of nuclei		Y	
	Voronoi		Y	
	Watershed		Y	
Deep learning-based	Cellpose	[2]	Y	
	JSTA	[21]	Y	
	GeneSegNet	[29]	N	Unclear how data should be processed into the required format and used to generate initial labels, missing instructions and/or code.
	SCS	[24]	N	No cells were detected for Xenium, CosMx, and MERSCOPE, and considerably lengthy runtimes compared to other methods (above 72-hour practical requirement) for Stereo-seq.
	Mesmer	[30]	N	Poor performance (neighbouring contamination); consistently rounded morphologies that do not demonstrate the diverse morphologies expected for the cell types.
Transcript-based	Baysor	[16]	Y	
	pciSeq	[20]	N	Method designed to assign transcripts to cells, does not consider cell boundaries and their overlap.
	ClusterMap	[22]	N	Poor performance (cells too large and few in number).
	Sparcle	[33]	N	Method designed to assign transcripts to cells, does not consider cell boundaries and their overlap.
	StereoCell	[18]	N	Poor performance (neighbouring contamination); consistently rounded morphologies that do not demonstrate the diverse morphologies expected for the cell types.

**Supplementary Table 2. Summary of all CellSPA evaluation metrics of segmentation aspects across five complementary categories.**			
**Metrics category**	**Metrics name**	**Gene/Cell/ Dataset level**	**Description**
Baseline	Number of cells (# cells)	Dataset level	
	Proportion of transcripts assigned	Dataset level	
	Proportion of cells expressed per gene	Gene level	
	Number of transcripts per cell	Cell level	
	Number of genes expressed per cell	Cell level	
	Cell area	Cell level	
	Density	Cell level	Number of total transcripts/Cell area
	Elongation	Cell level	Ratio between length and Width of objects bounding box
	Circularity	Cell level	Ratio of the area of an object to the area of a circle with the same convex perimeter
	Sphericity	Cell level	Degree to which object approaches shape of sphere
	Compactness	Cell level	Ratio of the area of an object to the area of a circle with the same perimeter
	Convexity	Cell level	Convexity is the ratio of an objects area to its convex area
	Eccentricity	Cell level	Ratio of the minor axis of an object to the major axis of an object
	Solidity	Cell level	Ratio of the area of an object to its convex area
Cell Expression	Average expression similarity	Cell type level	Calculate association between average expression profile for each cell type from segmented cells and scRNA-seq data.
	proportion of non-zero expression similarity	Cell type level	Calculate association between cell type % expressed profile from segmented cells and scRNA-seq data.
	Cell type proportion similarity	Dataset level	Calculating correlation with cell type proportion in paired Chromium data
	Positive markers purity F1	Cell level	
	Positive markers purity precision	Cell level	
	Positive markers purity recall	Cell level	
	Positive markers expressed %	Cell level	
	Negative markers purity F1	Cell level	
	Negative markers purity precision	Cell level	
	Negative markers purity recall	Cell level	
	Negative markers expressed %	Cell level	
Spatial characteristics (Association between cell type diversity with cell-level baseline characteristics)	corr - CTDiversity x CV(Num cell)	Cell type level	Pearson correlation between cell type diversity and coefficient of variation of Number of cells
	corr - CTDiversity x CV(Prop of transcripts)	Cell type level	Correlation between cell type diversity and coefficient of variation of Proportion of transcripts assigned
	corr - CTDiversity x CV(Prop of cells per gene)	Cell type level	Correlation between cell type diversity and coefficient of variation of Proportion of cells expressed per gene

	corr - CTDiversity x CV(Num transcripts per cell)	Cell type level	Correlation between cell type diversity and coefficient of variation of Number of transcripts per cell
	corr - CTDiversity x CV(Num genes per cell)	Cell type level	Correlation between cell type diversity and coefficient of variation of Number of genes expressed per cell
	corr - CTDiversity x CV(Cell area)	Cell type level	Correlation between cell type diversity and coefficient of variation of Cell area
	corr - CTDiversity x CV(Density)	Cell type level	Correlation between cell type diversity and coefficient of variation of Density
	corr - CTDiversity x CV(Elongation)	Cell type level	Correlation between cell type diversity and coefficient of variation of Elongation
	corr - CTDiversity x CV(Circularity)	Cell type level	Correlation between cell type diversity and coefficient of variation of Circularity
	corr - CTDiversity x CV(Sphericity)	Cell type level	Correlation between cell type diversity and coefficient of variation of Sphericity
	corr - CTDiversity x CV(Compactness)	Cell type level	Correlation between cell type diversity and coefficient of variation of Compactness
	corr - CTDiversity x CV(Convexity)	Cell type level	Correlation between cell type diversity and coefficient of variation of Convexity
	corr - CTDiversity x CV(Eccentricity)	Cell type level	Correlation between cell type diversity and coefficient of variation of Eccentricity
	corr - CTDiversity x CV(Solidity)	Cell type level	Correlation between cell type diversity and coefficient of variation of Solidity
Nearest Neighbour interaction	Percentage of negative markers expressed in neighbour (unwanted expression)	Dataset level	For a pair of cell type, calculate the negative marker expressed proportion of cell type A vs the distance to cell type B
Robustness and reproducibility between two biological replicates	Concordance between Number of cells	Dataset level	Correlation between Number of cells
	Concordance between Proportion of transcripts assigned	Dataset level	Correlation between Proportion of transcripts assigned
	Concordance between Proportion of cells expressed per gene	Dataset level	Correlation between Proportion of cells expressed per gene
	Concordance between Number of transcripts per cell	Dataset level	Correlation between Number of transcripts per cell
	Concordance between Number of genes expressed per cell	Dataset level	Correlation between Number of genes expressed per cell
	Concordance between Cell area	Dataset level	Correlation between Cell area
	Concordance between Density	Dataset level	Correlation between Density
	Concordance between Elongation	Dataset level	Correlation between Elongation
	Concordance between Circularity	Dataset level	Correlation between Circularity
	Concordance between Sphericity	Dataset level	Correlation between Sphericity
	Concordance between Compactness	Dataset level	Correlation between Compactness
	Concordance between Convexity	Dataset level	Correlation between Convexity
	Concordance between Eccentricity	Dataset level	Correlation between Eccentricity
	Concordance between Solidity	Dataset level	Correlation between Solidity
* correlation by default is defined as Pearson correlation			

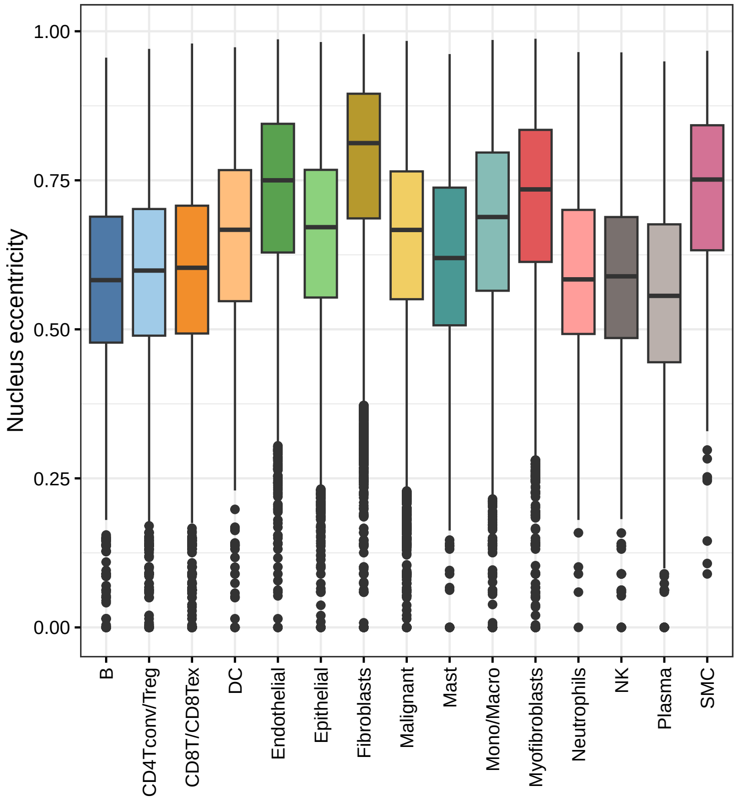

Supplementary Figure 1: Boxplots showing the eccentricity of nuclei for different cell types for Xenium-BreastCancer1. The number points for each box includes the number of cells detected by each cell type (N = B: 4656; CD4Tconv/Treg: 7738; CD8T/CD8Tex:10923; DC: 2074; Endothelial: 6107; Epithelial:11391; Fibroblasts:14354; Malignant:28697; Mast: 1047; Mono/Macro: 8430; Myofibroblasts: 7925; Neutrophils: 680; NK: 1237; Plasma: 1986; SMC: 567), ranges from the first to third quartile with the median as the horizontal line. The box plot's lower whisker extends 1.5 times the interquartile range below the first quartile, while the upper whisker extends 1.5 times the interquartile range above the third quartile. Source data are provided as a Source Data file.

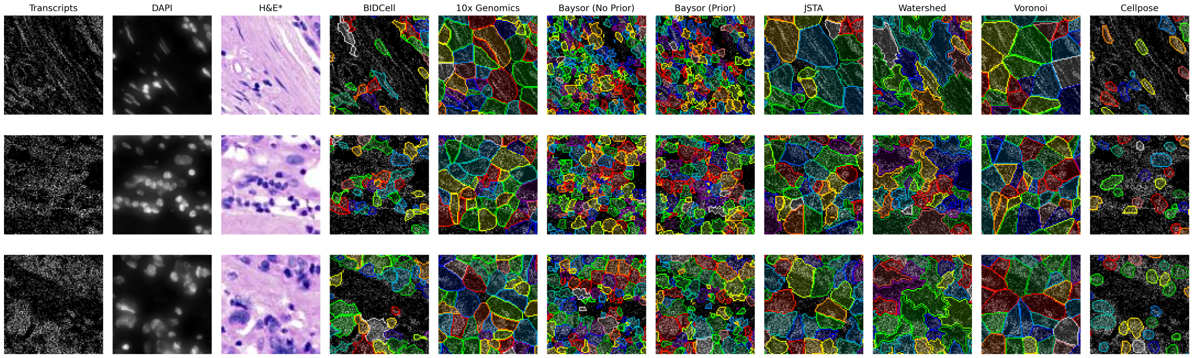

(a) Xenium-BreastCancer1

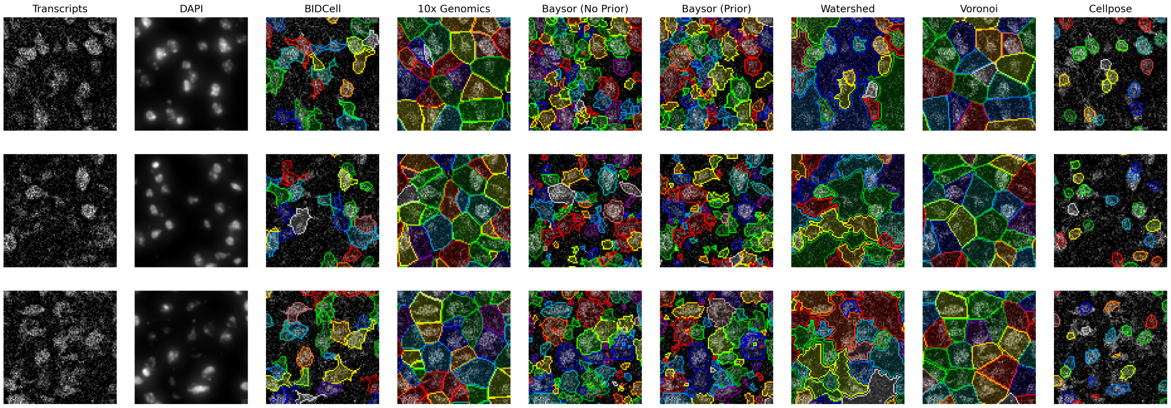

(b) Xenium-MouseBrain

Supplementary Figure 2: Further selective illustration of BIDCell and other segmentation methods on (a) Xenium-BreastCancer1 and (b) Xenium-MouseBrain. H&E images are shown for visualisation purposes only. BIDCell generate morphologies that exhibit better visual correspondence with the input images.

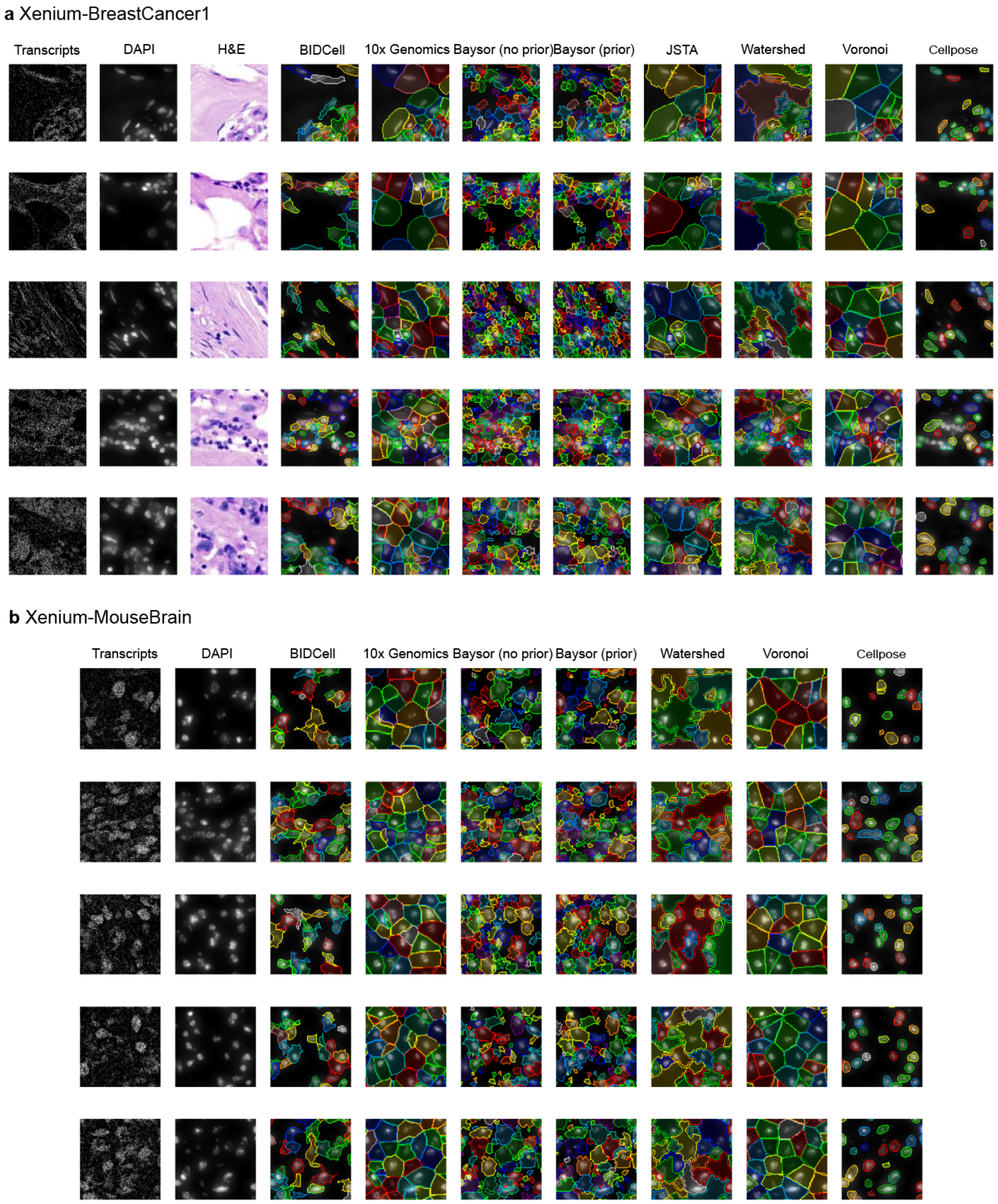

Supplementary Figure 3: Illustration of BIDCell and other segmentation methods overlaid on DAPI images for (a) Xenium-BreastCancer1 and (b) Xenium-MouseBrain.

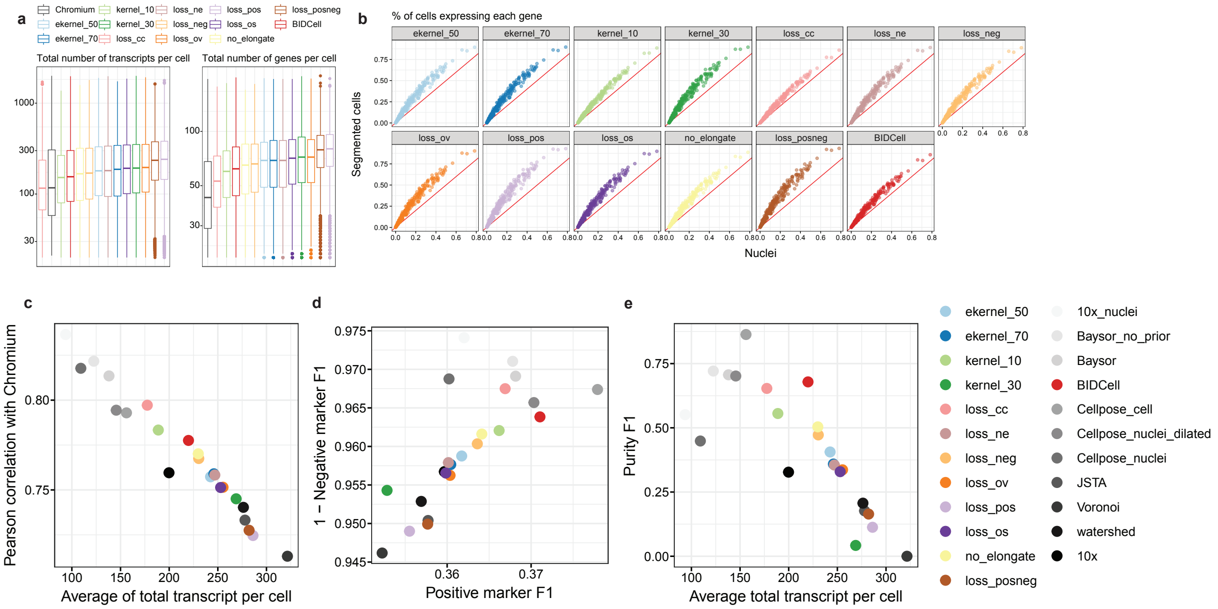

Supplementary Figure 4: Ablation study of different settings of main parameters: `ekernel_50` and `ekernel_70`, where l_t of the elliptical kernel in the cell-calling loss was 50 or 70; `kernel_10` and `kernel_30`, where the diameter of the circular kernel in the cell-calling loss was 10 and 30; `loss_`, where each loss was individually set to zero; `no_elongate`, where the ability to predict elongated cells is removed; and `BIDCell`, the proposed settings. (a) Box plots of the number of transcripts and genes per cell for the different settings. The number points for each box includes the number of cells detected by each method (N = Chromium: 22294; BIDCell: 103209; `ekernel_50`: 102740; `ekernel_70`: 102262; `kernel_10`: 102124; `kernel_30`: 101211; loss_cc: 100793; loss_ne: 101961; loss_neg: 102264; loss_ov: 103027; loss_pos: 105368; loss_os: 102700; no_elongate: 101873), ranges from the first to third quartile with the median as the horizontal line. The box plot's lower whisker extends 1.5 times the interquartile range below the first quartile, while the upper whisker extends 1.5 times the interquartile range above the third quartile; (b) scatter plot showing genes expressed in the segmented cells compared to the nuclei; (c) scatter plot between Pearson correlation with Chromium (y-axis) and total transcripts per cell (x-axis); (d) scatter plot between inverse negative marker F1 (y-axis) and positive marker F1 (x-axis); and (e) scatter plot between purity F1 (y-axis) and total transcripts per cell (x-axis). All the loss functions except the cell-calling loss increased the expression purity. The cell-calling loss increased the number of transcripts captured, without detriment to expression purity. The nucleus encapsulation loss improved purity metrics, by leveraging genes expressed in nuclei to guide the capture of genes expressed in cells. The overlap and over-segmentation losses improve the shapes of predicted cells, which translates into improved purity metrics. The differences between `BIDCell` and `loss_posneg` show that the marker losses improved performance considerably, particularly in purity metrics and Pearson correlation to Chromium data, while more transcripts were captured in the segmented cells. Source data are provided as a Source Data file.

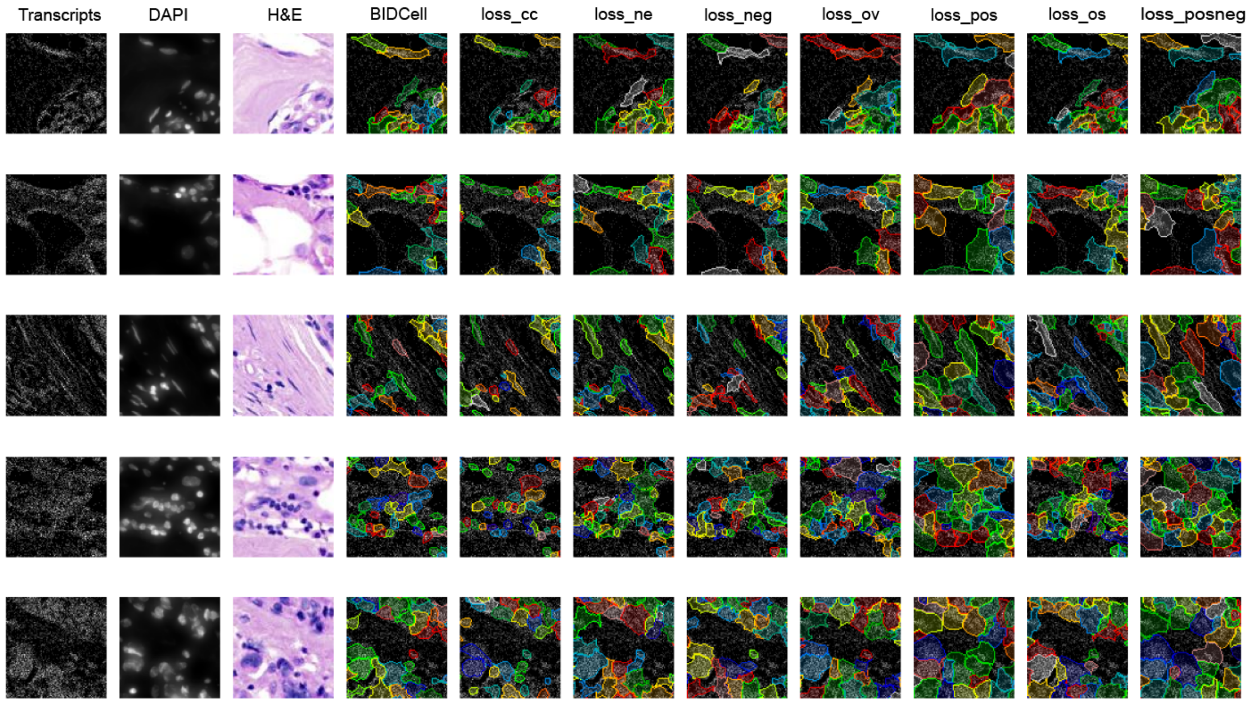

Supplementary Figure 5: Illustrations of the segmentation predictions from different ablation experiments, where each loss was individually set to zero as indicated by `loss_`. The segmentations without cell-calling loss tend to be too small, while they are too large when marker losses are excluded. Exclusion of the other losses reduced the correspondence of the cell boundaries to the input images.

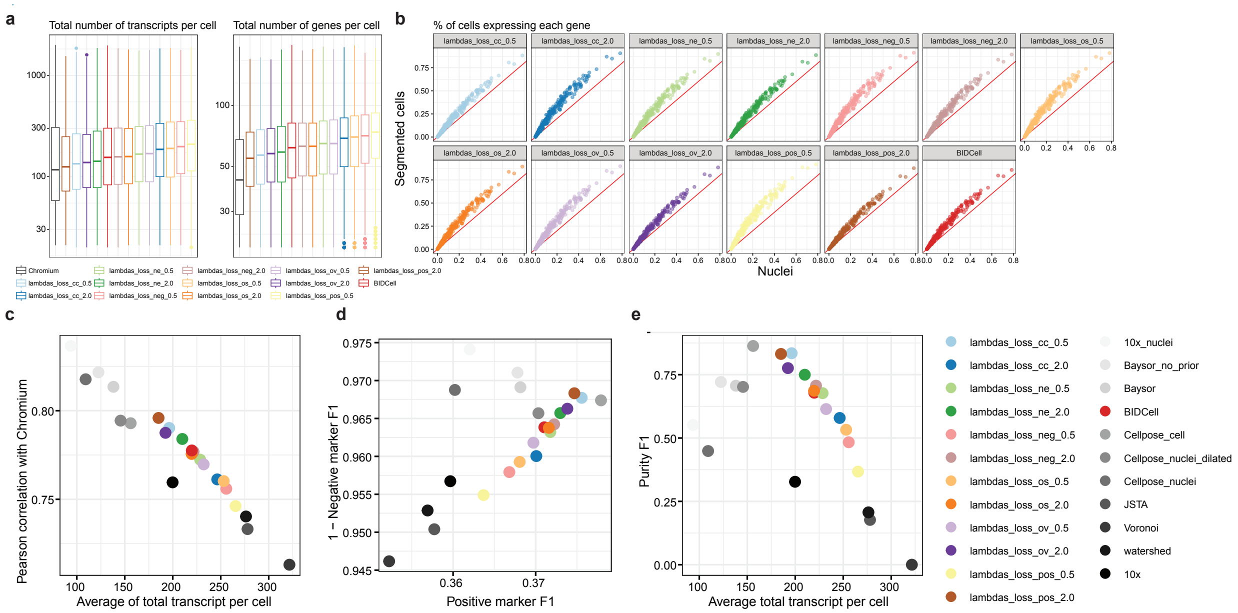

Supplementary Figure 6: Performance of BIDCell with different weights (lambdas) for each loss function. (a) Box plots of the number of transcripts and genes per cell for the different settings. The number points for each box includes the number of cells detected by each method (N = Chromium: 22294; BIDCell: 103209; lambdas_loss_cc_0.5: 102917; lambdas_loss_cc_2.0: 104158; lambdas_loss_ne_0.5: 103843; lambdas_loss_ne_2.0: 103129; lambdas_loss_neg_0.5: 104327; lambdas_loss_neg_2.0: 103543; lambdas_loss_os_0.5: 103975; lambdas_loss_os_2.0: 103739; lambdas_loss_ov_0.5: 103847; lambdas_loss_ov_2.0: 103063; lambdas_loss_pos_0.5: 104558; lambdas_loss_pos_2.0: 102724), ranges from the first to third quartile with the median as the horizontal line. The box plot's lower whisker extends 1.5 times the interquartile range below the first quartile, while the upper whisker extends 1.5 times the interquartile range above the third quartile; (b) scatter plot showing genes expressed in the segmented cells compared to the nuclei; (c) scatter plot between Pearson correlation with Chromium (y-axis) and total transcripts per cell (x-axis); (d) scatter plot between inverse negative marker F1 (y-axis) and positive marker F1 (x-axis); and (e) scatter plot between purity F1 (y-axis) and total transcripts per cell (x-axis). BIDCell performs well across various datasets and technologies when using default weights for all losses (set to 1.0), and metrics may be further improved by setting different weights. Pearson correlation and purity metrics were higher with higher weights for positive marker, nuclei, and overlap losses, and lower weights for the cell-calling loss. Source data are provided as a Source Data file.

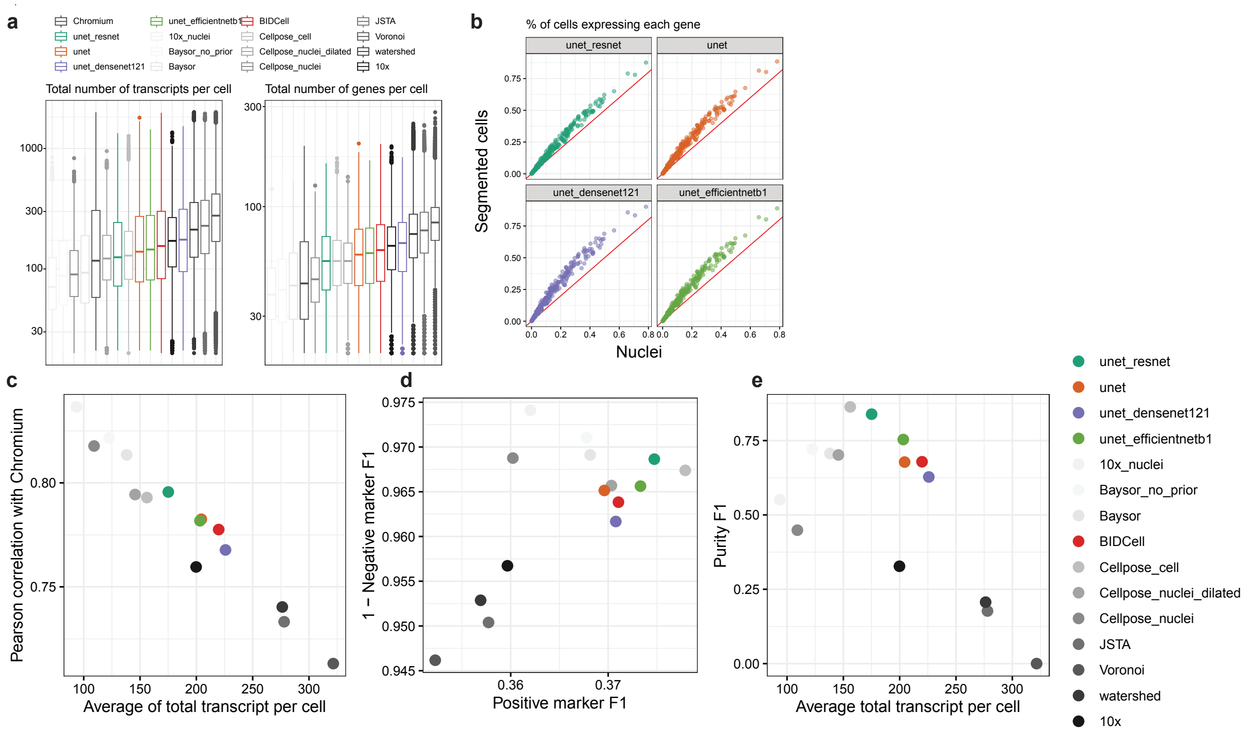

Supplementary Figure 7: Performance of BIDCell with different backbones (U-Net, U-Net-ResNet18, U-Net-DenseNet121, and U-Net-EfficientNetB1). These results further demonstrate the flexibility of our BIDCell framework with alternative backbones, as it achieved consistently high performance, with relatively high purity and additional transcript information to the nuclei. (a) Box plots of the number of transcripts and genes per cell for the different settings. The number points for each box includes the number of cells detected by each method (N = Chromium: 22294; Cellpose (nuclei): 99693; BIDCell: 103209; 10x (nulcei): 126515; 10x: 160254; JSTA: 107131; Cellpose_nuclei_dilated: 104307; Cellpose_cell: 87046; Voronoi: 106227; watershed: 105527; Baysor: 177437; Baysor_no_prior: 191698; U-Net: 102975; U-Net-ResNet18: 102659; U-Net-DenseNet121: 103757; U-Net-EfficientNetB1: 103009), ranges from the first to third quartile with the median as the horizontal line. The box plot's lower whisker extends 1.5 times the interquartile range below the first quartile, while the upper whisker extends 1.5 times the interquartile range above the third quartile; (b) scatter plot showing genes expressed in the segmented cells compared to the nuclei; (c) scatter plot between Pearson correlation with Chromium (y-axis) and total transcripts per cell (x-axis); (d) scatter plot between inverse negative marker F1 (y-axis) and positive marker F1 (x-axis); and (e) scatter plot between purity F1 (y-axis) and total transcripts per cell (x-axis). Source data are provided as a Source Data file.

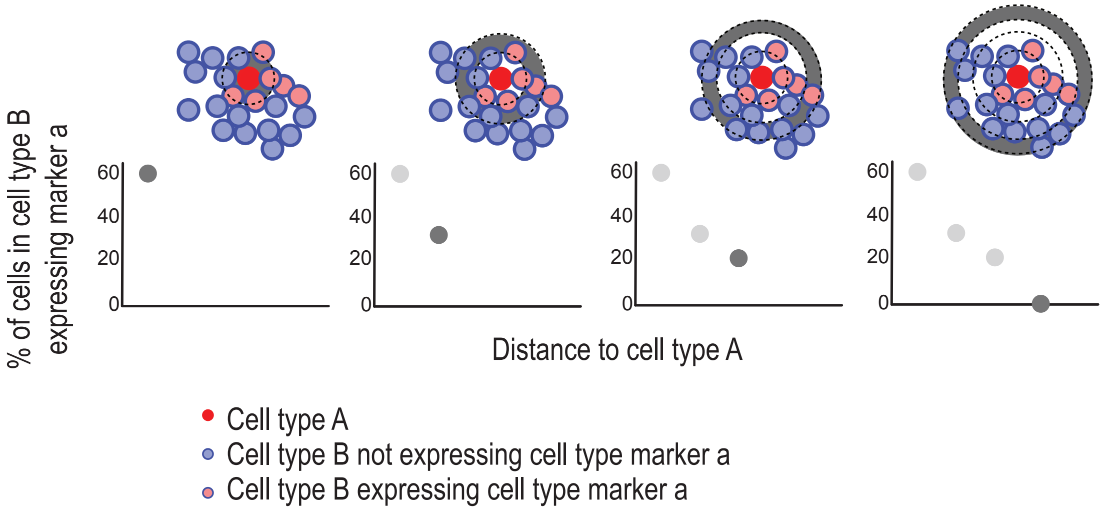

Supplementary Figure 8: Schematic figure for neighbouring contamination. A pair of cell type A and B and cell type A's marker a is predefined. For each cell type B, the distance to the nearest cell type A is calculated. The cell of cell type B is then grouped based on their distance to the nearest cell of cell type A (4 groups in the figure). The proportion of cells expressed marker a is then calculated.

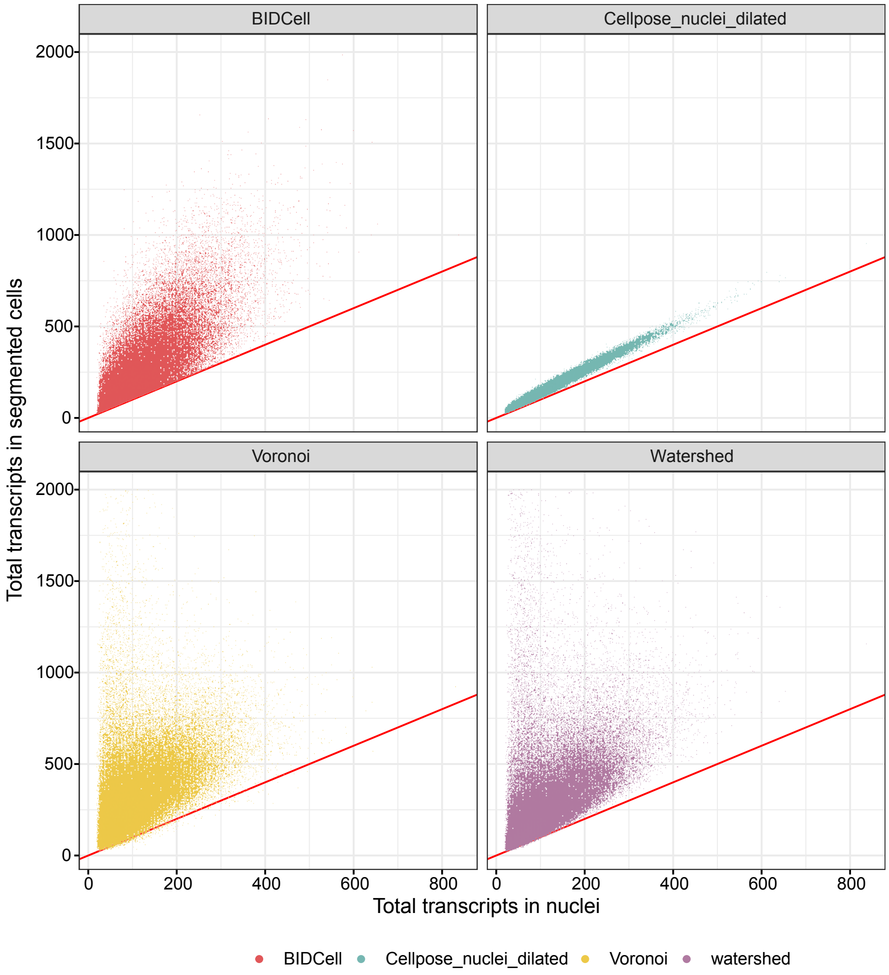

Supplementary Figure 9: Total transcripts per cell for Xenium-BreastCancer1, comparing between Cellpose nuclei and cells segmented using four methods (BIDCell, Cellpose nuclei dilated, Voronoi, and Watershed). The red diagonal line indicates where the total number of transcripts in the nuclei is equal to the number of transcripts in the segmented cells.

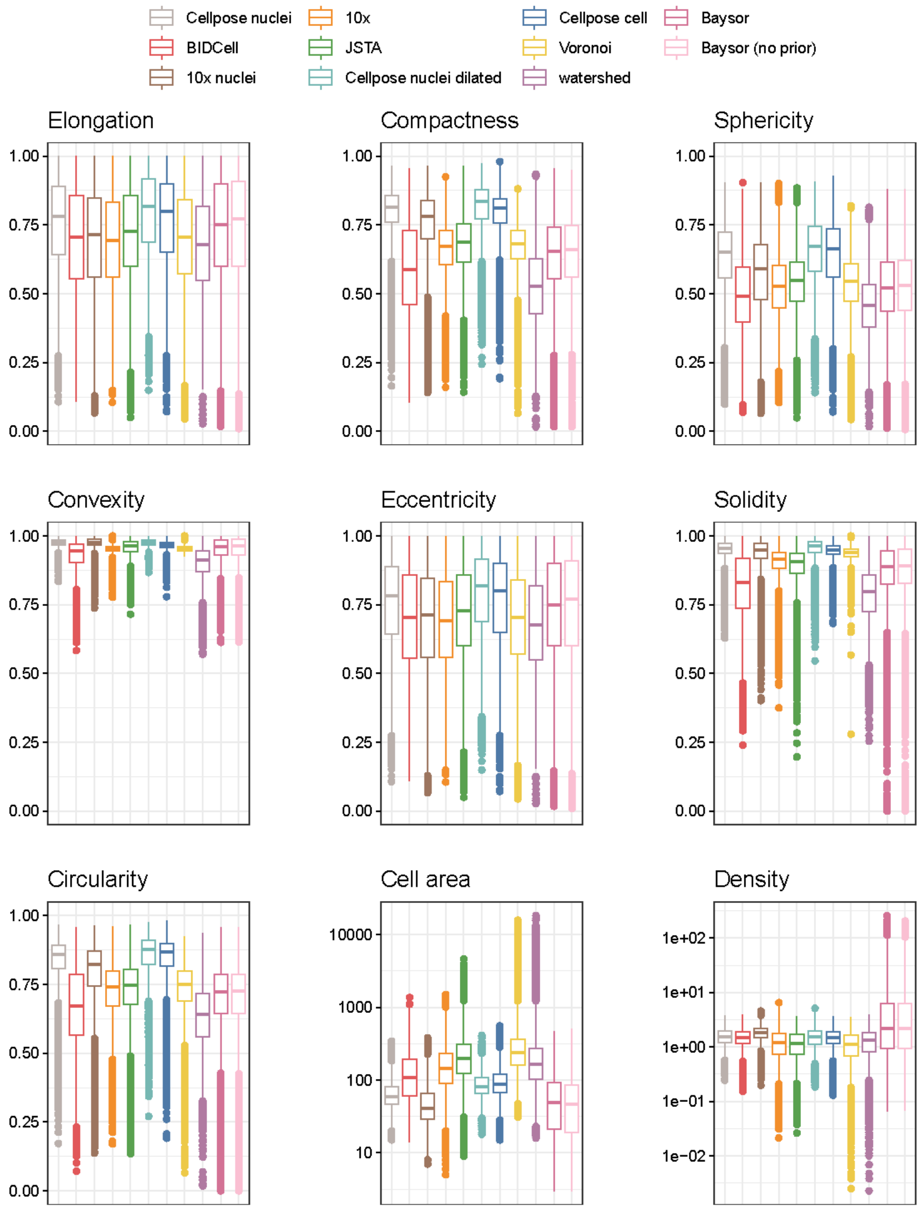

Supplementary Figure 10: Cell morphology metrics (elongation, compactness, sphericity, convexity, eccentricity, solidity, circularity, cell area, and density) of Xenium-BreastCancer1 for different cell segmentation methods. The number points for each box includes the number of cells detected by each method (N = Cellpose (nuclei): 99693; BIDCell: 103209; 10x (nulcei): 126515; 10x: 160254; JSTA: 107131; Cellpose_nuclei_dilated: 104307; Cellpose_cell: 87046; Voronoi: 106227; watershed: 105527; Baysor: 177437; Baysor_no_prior: 191698), ranges from the first to third quartile with the median as the horizontal line. The box plot's lower whisker extends 1.5 times the interquartile range below the first quartile, while the upper whisker extends 1.5 times the interquartile range above the third quartile.

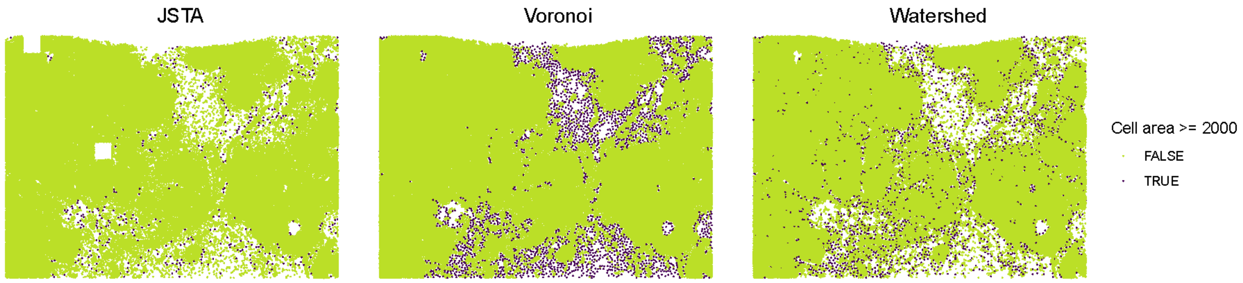

Supplementary Figure 11: Cell area outlier in three methods (JSTA, Voronoi, and Watershed), where we observe that these methods tend to produce cells with large areas in the sparse regions.

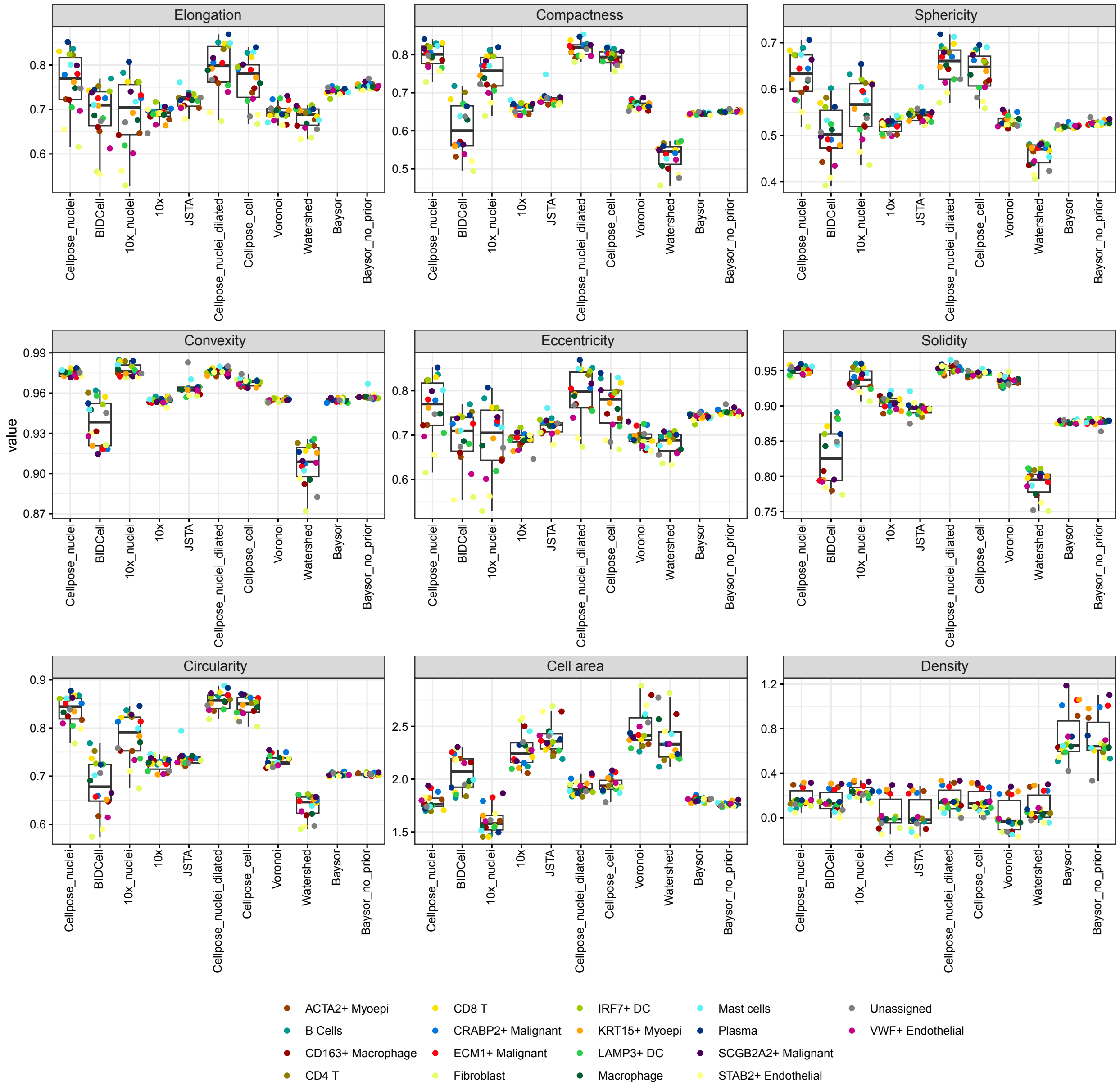

Supplementary Figure 12: Boxplots of average cell morphology metrics (elongation, compactness, sphericity, convexity, eccentricity, solidity, circularity, cell area, and density) per cell type of Xenium-BreastCancer1 for different cell segmentation methods, where each point indicates one cell type. The number points for each box includes the number of cell types (N = 18), ranges from the first to third quartile with the median as the horizontal line.

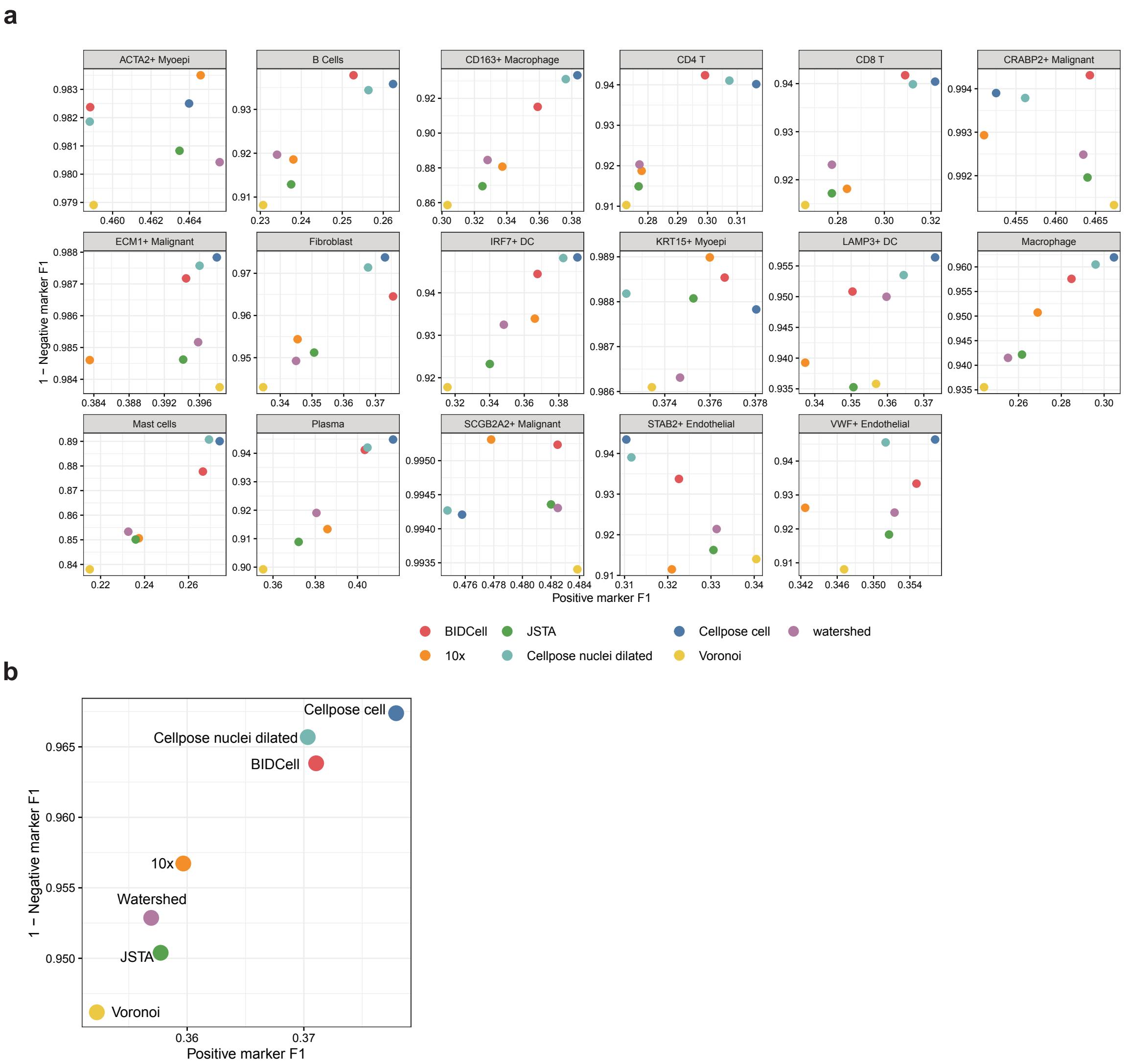

Supplementary Figure 13: Expression purity benchmarking results for Xenium-BreastCancer1. (a) Scatter plots showing the positive marker F1 scores vs. the 1 - negative marker F1 scores for each of the cell type, where each dot is one method. (b) Scatter plots showing the overall positive marker F1 vs. the 1 - negative marker F1. Source data are provided as a Source Data file.

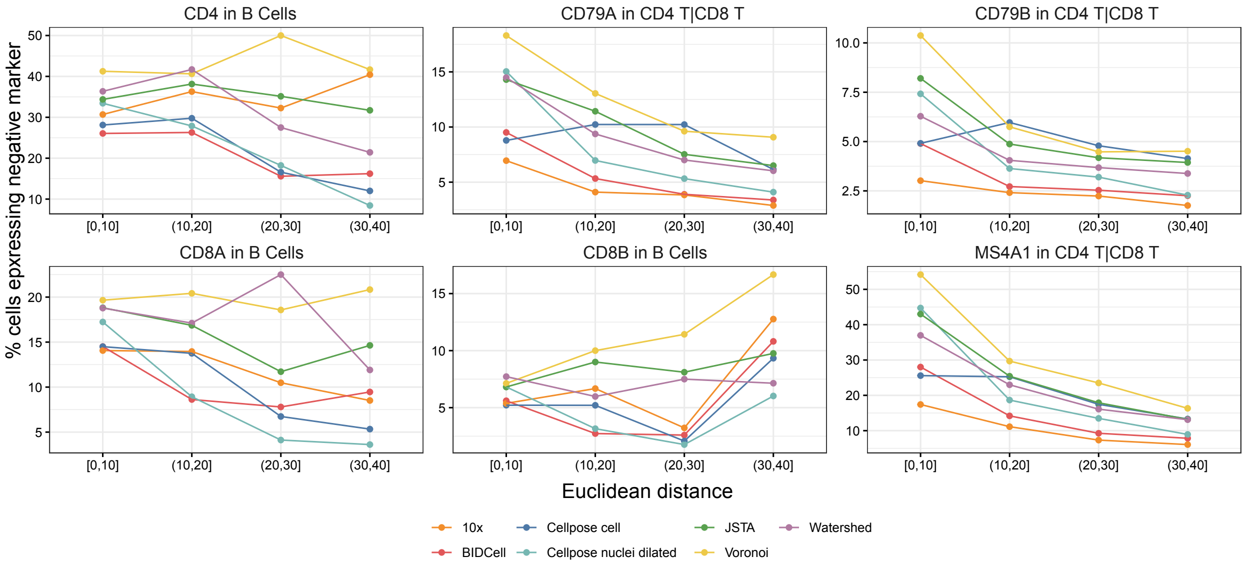

Supplementary Figure 14: Neighbouring contamination results for Xenium-BreastCancer1. The line plots indicate the percentage of B cells expressing the unwanted T cell marker CD4, CD8A, and CD8B against its distance from the nearest T cell, where the cells are grouped by a certain distance range; and the percentage of T cells expressing CD79A, CD79B, and MS4A1, against the distance from the nearest B cells. A lower percentage is better, and each line represents a different method. Source data are provided as a Source Data file.

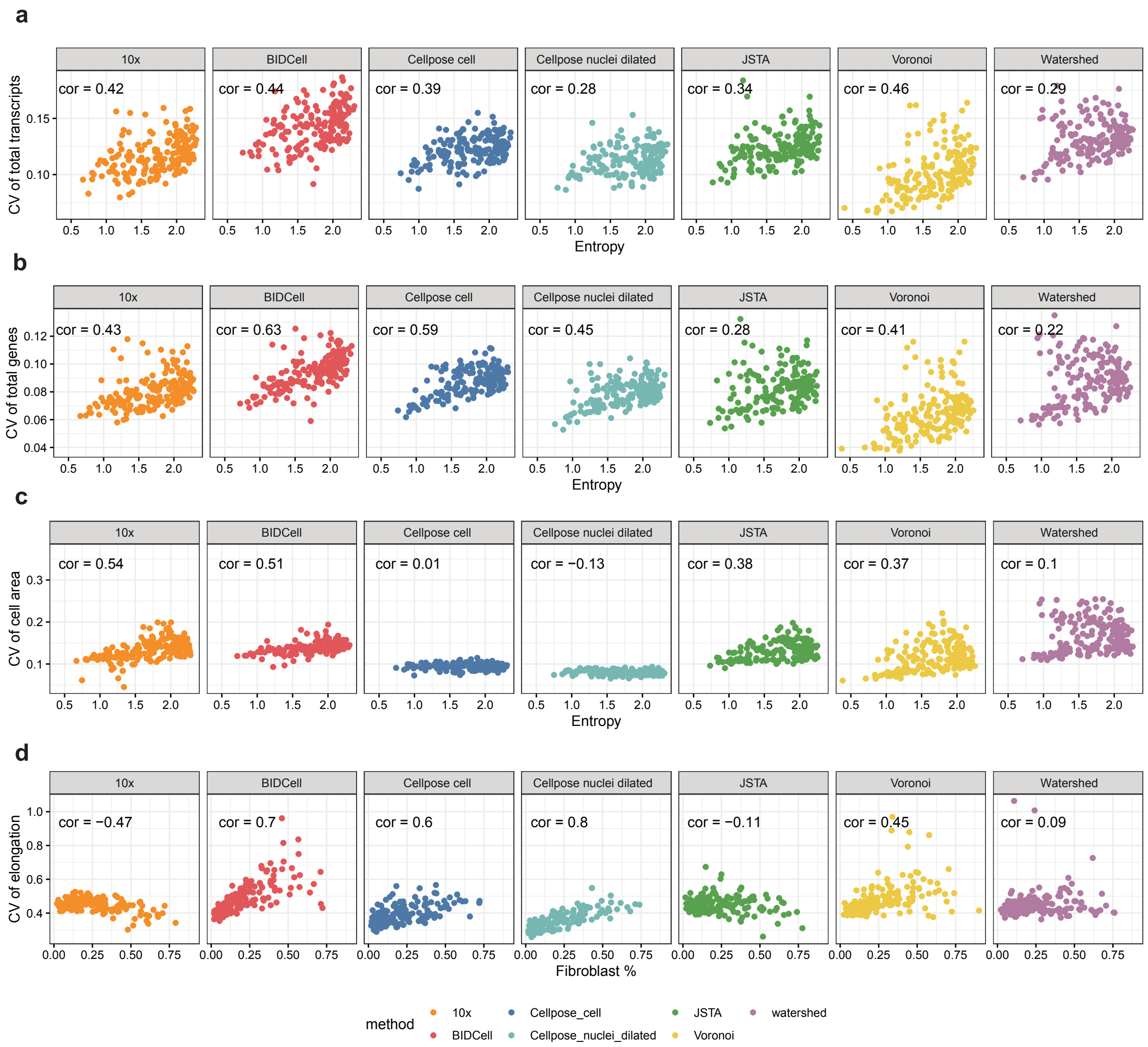

Supplementary Figure 15: Spatial diversity results for Xenium-BreastCancer1, corresponding to Figure 3g-h. Scatter plots showing the association between cell type entropy and (a) coefficient of variation of the total transcripts; (b) coefficient of variation of the total genes; and (c) coefficient of variation of cell area. (d) Scatter plots showing the association between the coefficient of variation of elongation and proportion of fibroblasts in the data. Source data are provided as a Source Data file.

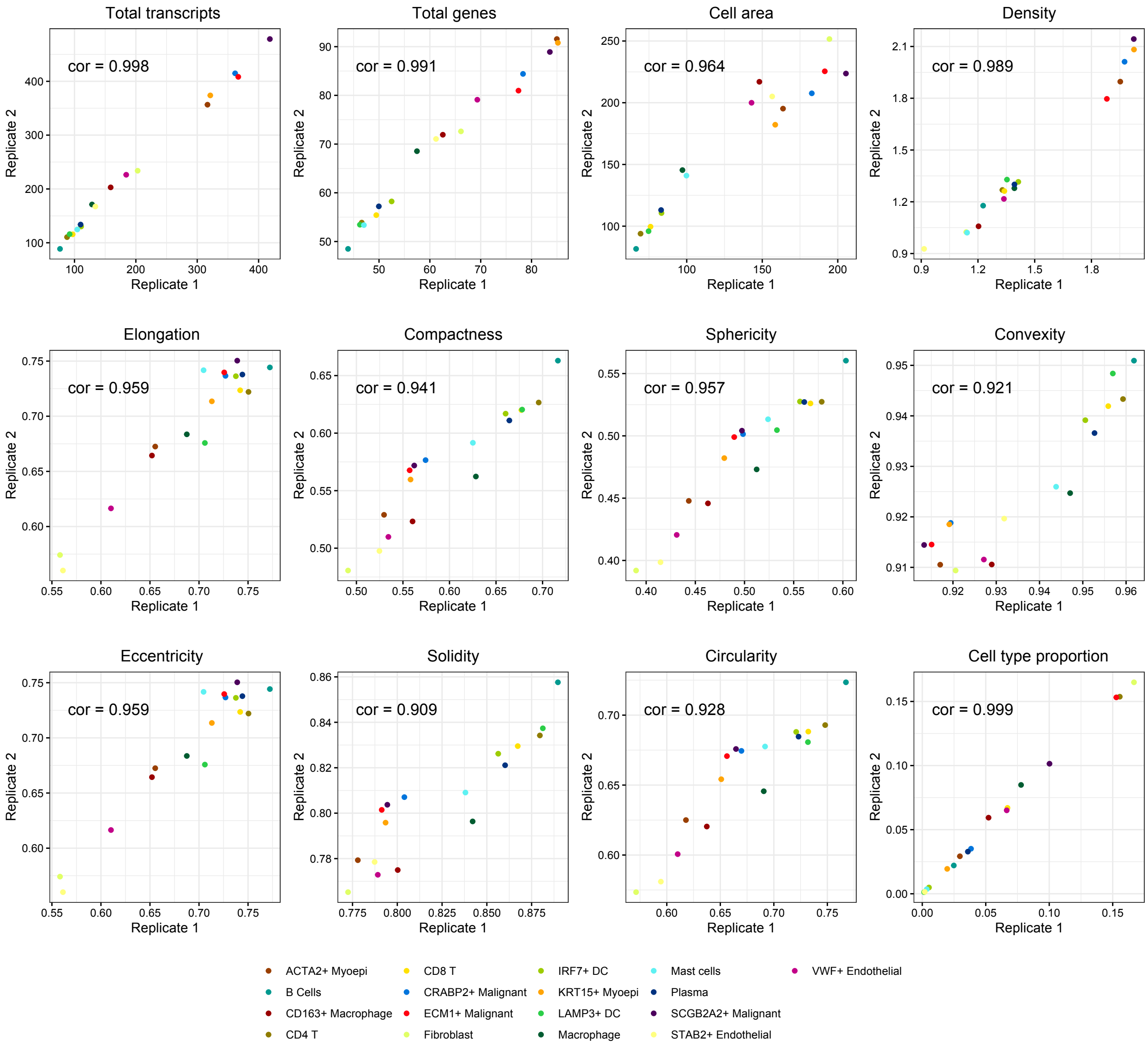

Supplementary Figure 16: Consistency between two replicates. A 3×4 scatter plots showing average cell-level baseline metrics, cell morphology metrics, and cell type proportions for cell types between two replicates. Source data are provided as a Source Data file.

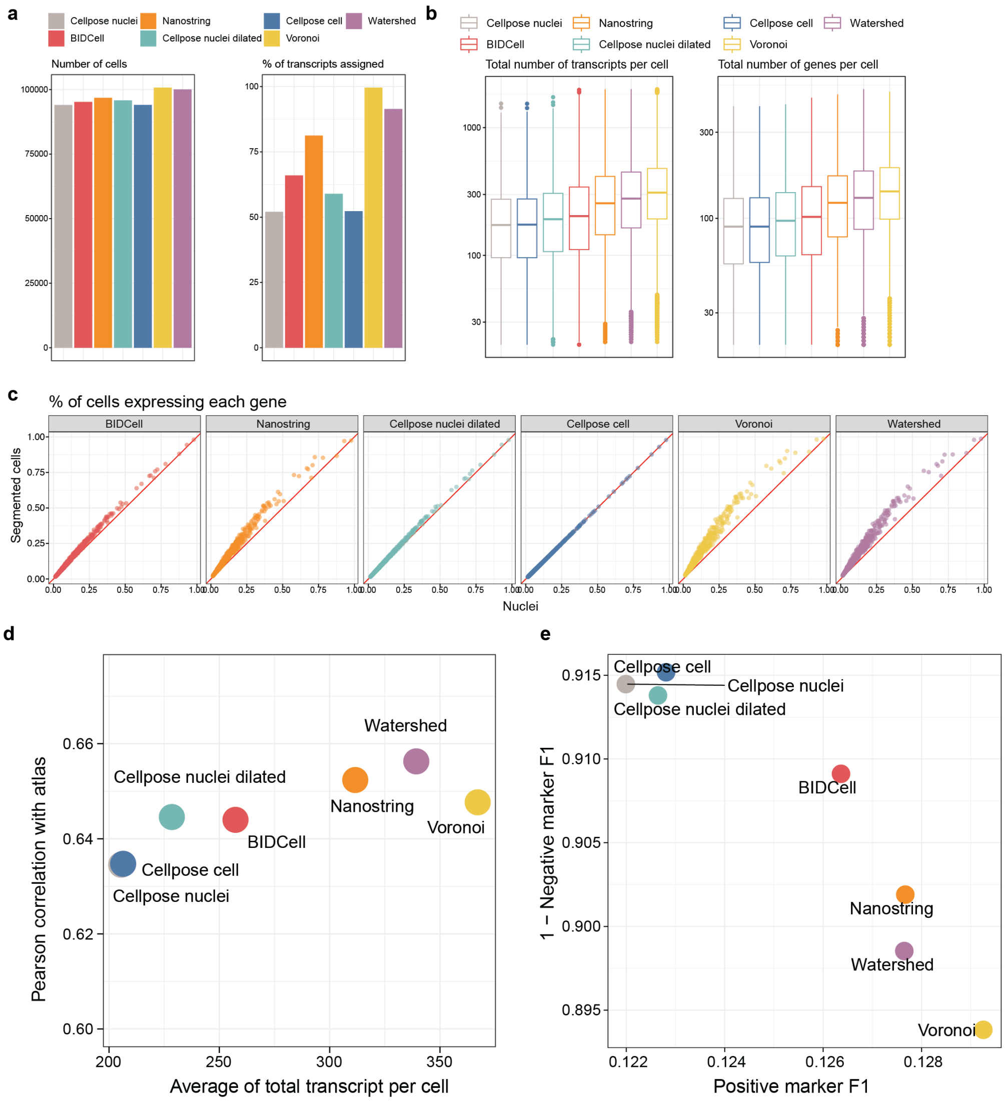

Supplementary Figure 17: Benchmarking results for CosMx-Lung. (a) Bar plot demonstrating overall characteristics, where the left panel shows the number of cells and the right panel shows the number of transcripts for each of the 7 methods. (b) Boxplot of cell-level quality metrics with total number of transcripts (left panel) and total number of genes (right panel). The number points for each box includes the number of cells detected by each method (N = Cellpose (nuclei): 94017; BIDCell: 95244; Nanostring: 96820; Cellpose_nuclei_dilated: 95849; Cellpose_cell: 94070; Voronoi: 100747; watershed: 100083), ranges from the first to third quartile with the median as the horizontal line. The box plot's lower whisker extends 1.5 times the interquartile range below the first quartile, while the upper whisker extends 1.5 times the interquartile range above the third quartile. (c) Gene-level quality metric represented by a scatter plot of percentage of cells expressed for each gene between the nuclei vs. the cell body. (d) Scatter plot between correlation with Chromium expression (y-axis) and average total number of transcripts per cell (x-axis), where each dot represents a different method. (e) Scatter plot between positive marker F1 score and 1 - negative marker F1 for each method. Source data are provided as a Source Data file.

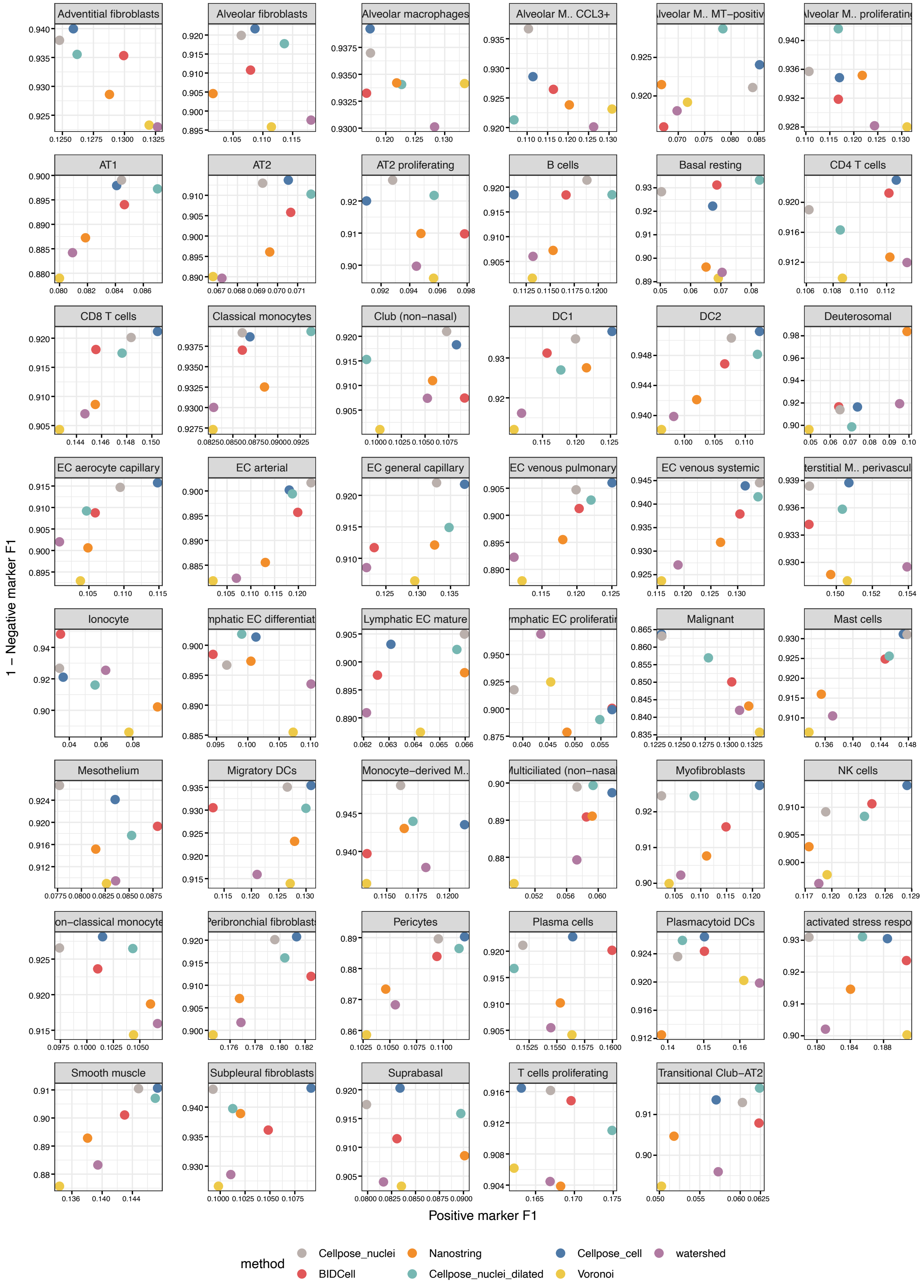

Supplementary Figure 18: Expression purity benchmarking results for CosMx-Lung: scatter plots showing the positive marker F1 scores vs. the 1 - negative marker F1 scores for each of the cell type, where each dot is one method. Source data are provided as a Source Data file.

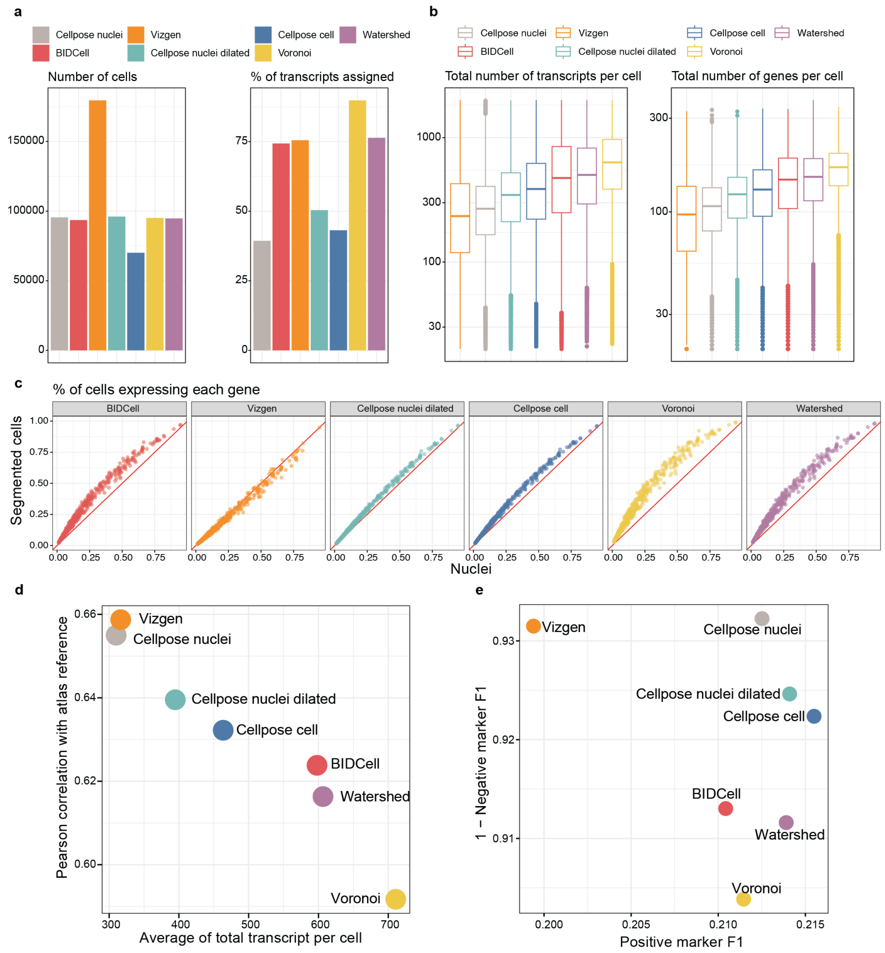

Supplementary Figure 19: Benchmarking results for MERSCOPE-Melanoma. (a) Bar plot demonstrating overall characteristics, where the left panel shows the number of cells and the right panel shows the number of transcripts for each of the 7 methods. (b) Boxplot of cell-level quality metrics with total number of transcripts (left panel) and total number of genes (right panel). (c) Gene-level quality metric represented by a scatter plot of percentage of cells expressed for each gene between the nuclei vs. the cell body. The number points for each box includes the number of cells detected by each method (N = Cellpose (nuclei): 95527; BIDCell: 93556; Vizgen: 179547; Cellpose_nuclei_dilated: 96059; Cellpose_cell: 70058; Voronoi: 95097; watershed: 94752), ranges from the first to third quartile with the median as the horizontal line. The box plot's lower whisker extends 1.5 times the interquartile range below the first quartile, while the upper whisker extends 1.5 times the interquartile range above the third quartile. (d) Scatter plot between correlation with Chromium expression (y-axis) and average total number of transcripts per cell (x-axis), where each dot represents a different method. (e) Scatter plot between positive marker F1 score and 1 - negative marker F1 for each method. Source data are provided as a Source Data file.

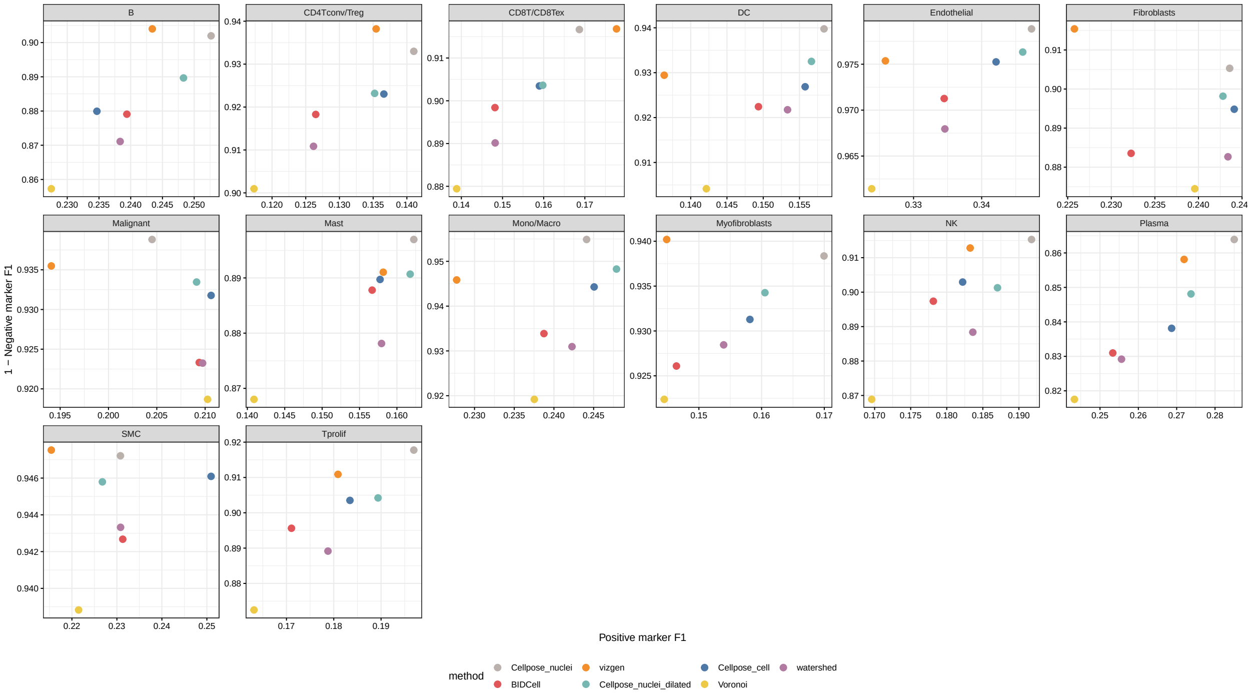

Supplementary Figure 20: Expression purity benchmarking results for MERSCOPE-Melanoma: scatter plots showing the positive marker F1 scores vs. the 1 - negative marker F1 scores for each of the cell type, where each dot is one method. Source data are provided as a Source Data file.

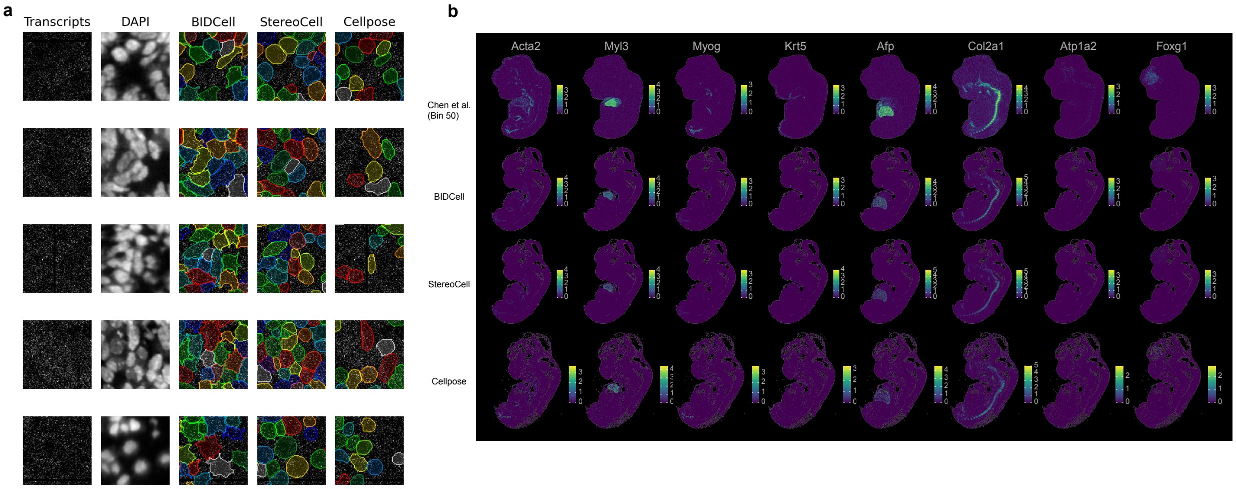

Supplementary Figure 21: Application of BIDCell to mouse embryo data from BGI Stereo-seq. (a) Example cell segmentations from BIDCell and StereoCell; and (b) examples of marker expression of spatial region for Chen et al. (Bin 50), BIDCell, and StereoCell.

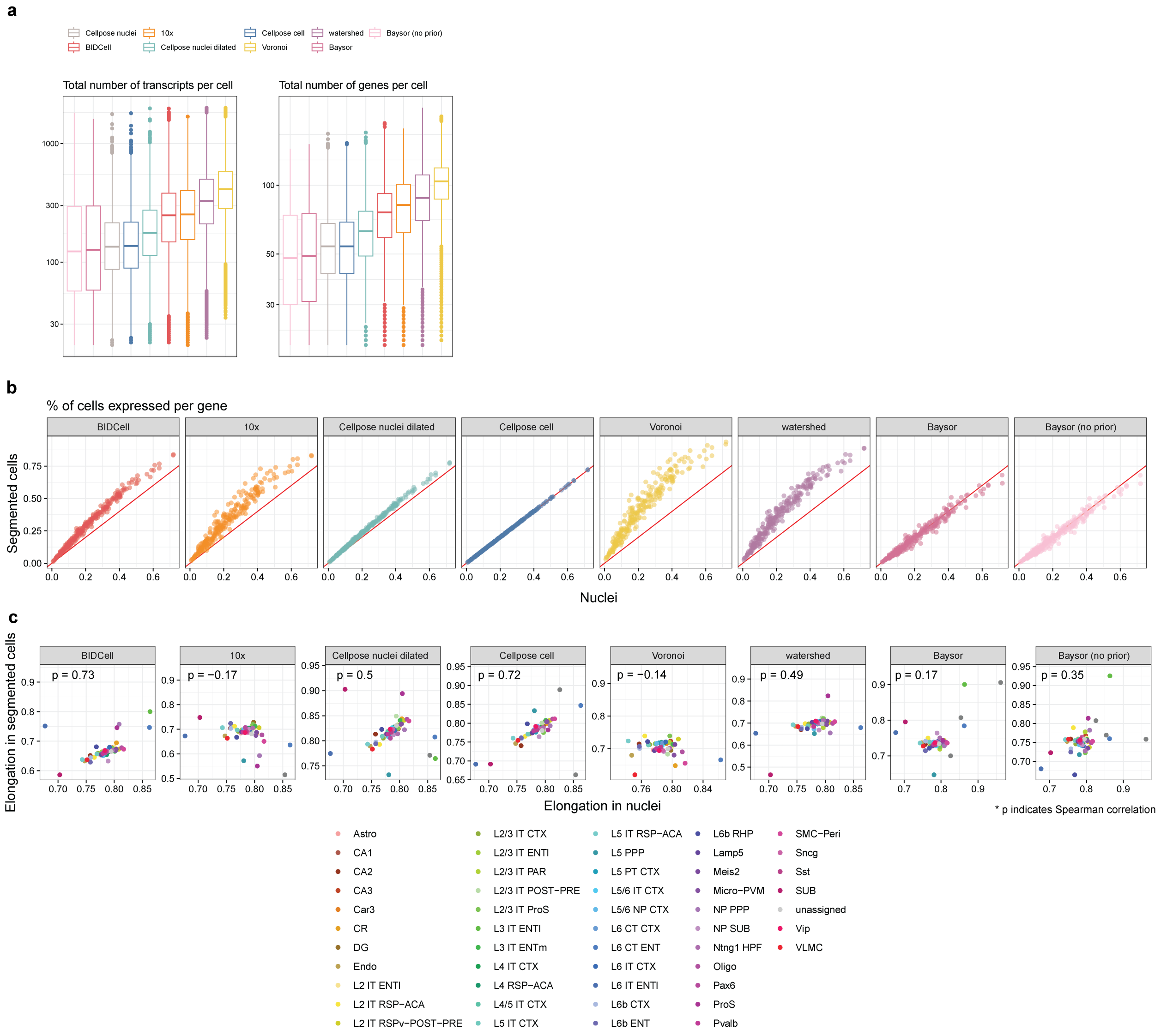

Supplementary Figure 22: Benchmarking results for Xenium-brain data. (a) Boxplot of cell-level quality metrics with total number of transcripts (left panel) and total number of genes (right panel). The number points for each box includes the number of cells detected by each method (N = Cellpose (nuclei):105279; BIDCell:107286; 10x:160994; Cellpose nuclei dilated:107360; Cellpose cell:105649; Voronoi:107910; watershed:107541; Baysor:175277; Baysor (no prior):161818), ranges from the first to third quartile with the median as the horizontal line. The box plot's lower whisker extends 1.5 times the interquartile range below the first quartile, while the upper whisker extends 1.5 times the interquartile range above the third quartile. (b) Gene-level quality metric represented by a scatter plot of percentage of cells expressed for each gene between the nuclei vs. the cell body. (c) Cell morphology metrics represented by the elongation values between the nuclei and segmented cell, where each dot represents the average elongation metrics for each cell type. Source data are provided as a Source Data file.

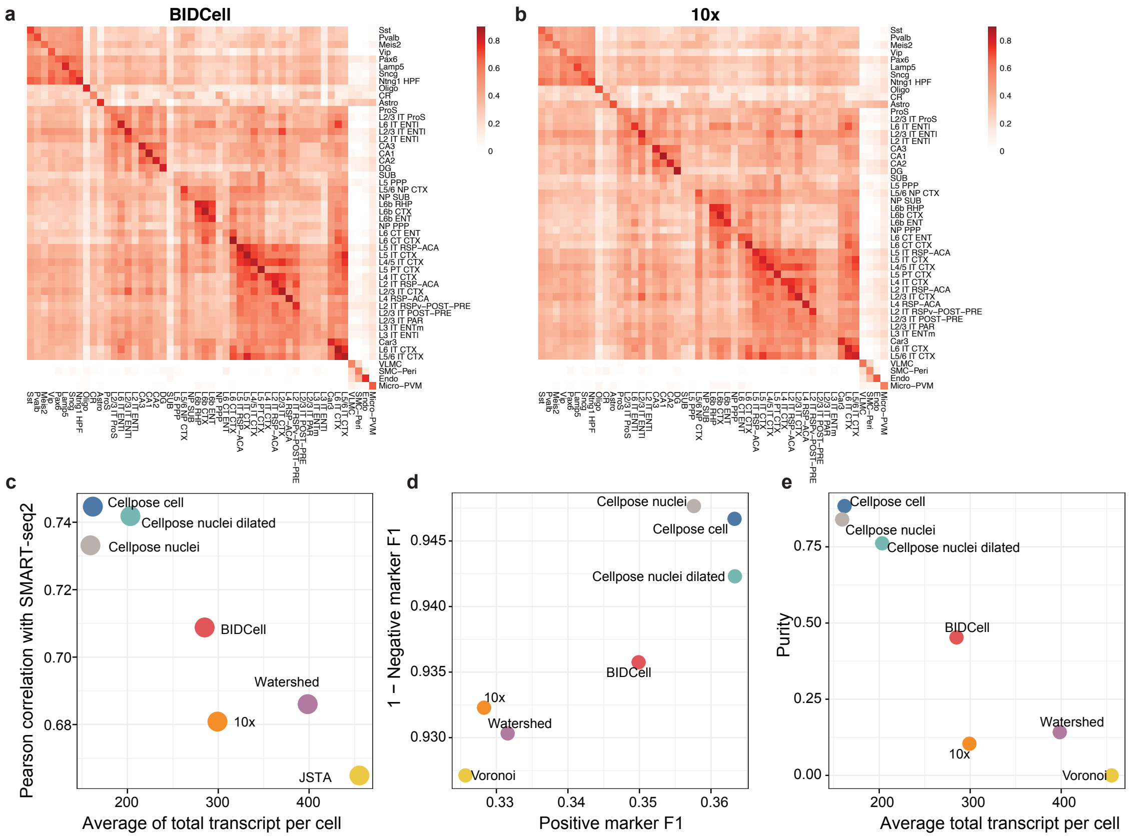

Supplementary Figure 23: Benchmarking results for Xenium-brain data. (a-b) Correlation heatmap of average expression between expression from Allan brain SMART-seq2 data (x-axis) and segmented cells from (a) BIDCell (y-axis); (b) 10x. (c) Scatter plot between correlation with Chromium expression (y-axis) and average total number of transcripts per cell (x-axis), where each dot represents a different method. (d) Scatter plot between positive marker F1 score and 1 - negative marker F1 for each method. (e) Scatter plot showing the purity F1 score against the average total transcripts per cell for each method. Source data are provided as a Source Data file.

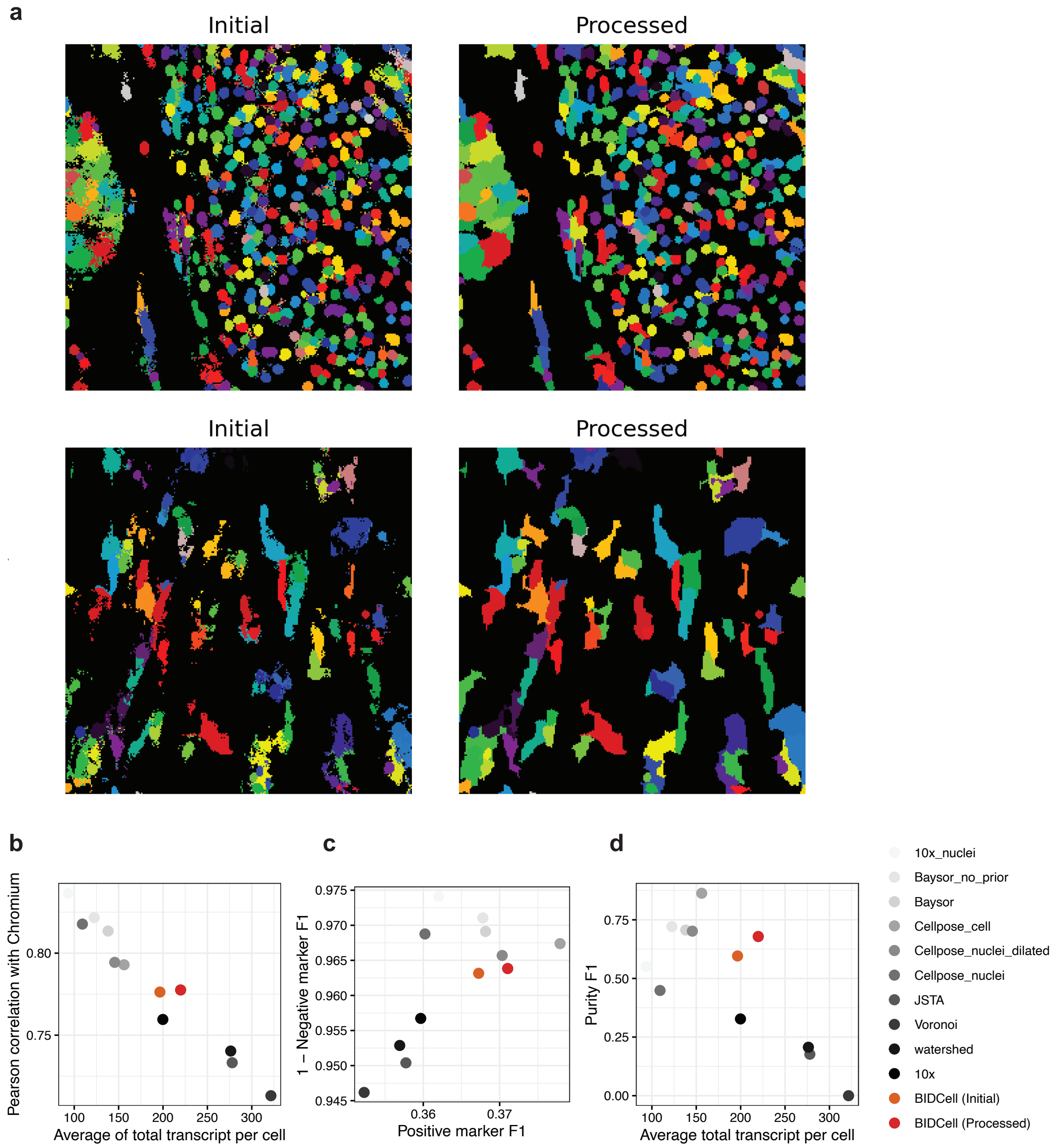

Supplementary Figure 24: Performance of BIDCell with or without morphological processing. (a) Example images to illustrate BIDCell before and after morphological processing; (b) scatter plot between Pearson correlation with Chromium (y-axis) and total transcripts per cell (x-axis); (c) scatter plot between inverse negative marker F1 (y-axis) and positive marker F1 (x-axis); and (d) scatter plot between purity F1 (y-axis) and total transcripts per cell (x-axis). Source data are provided as a Source Data file.

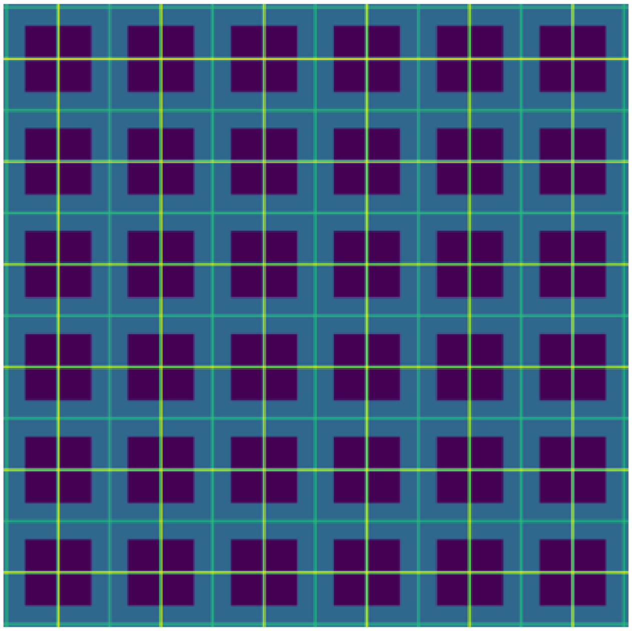

Supplementary Figure 25: Illustration of the aggregation of patches to minimise border effects. When dividing the transcriptomic maps into patches, we create two sets of patches of the same lateral dimensions with an overlap equal to half the lateral size of the patches. The first set (green borders) is obtained from a straightforward grid partitioning, while the second set (yellow border) overlaps the first set. The final prediction comprises the purple regions from the first set, and the blue regions from the second set.

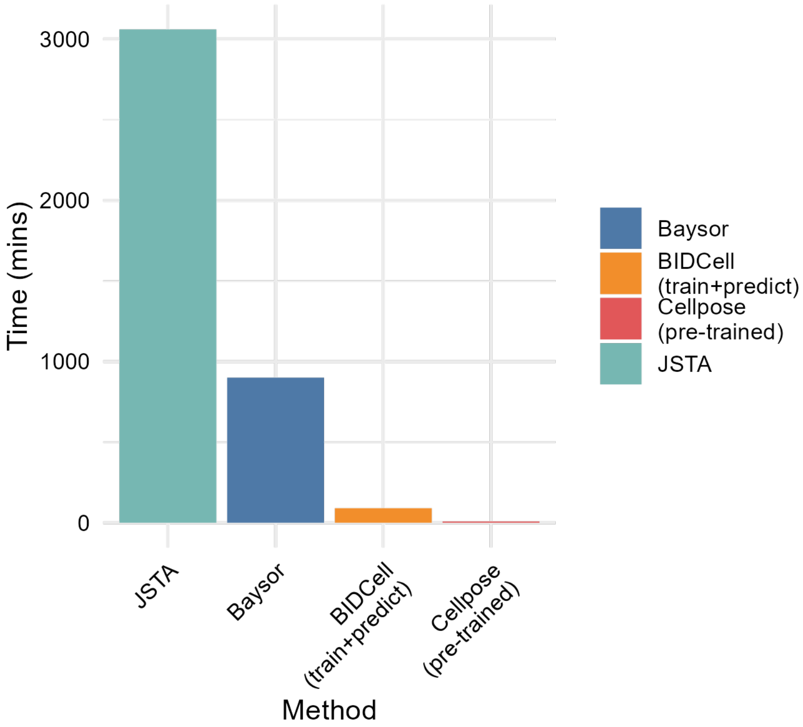

Supplementary Figure 26: Bar plot showing a comparison of the runtimes of different methods when applied to Xenium-BreastCancer1 (34 million transcripts). BIDCell achieved superior performance with a reasonable runtime. We note that we used a GPU with 12GB VRAM. BIDCell will run faster on GPUs with more VRAM, which is commonly at least 24GB.

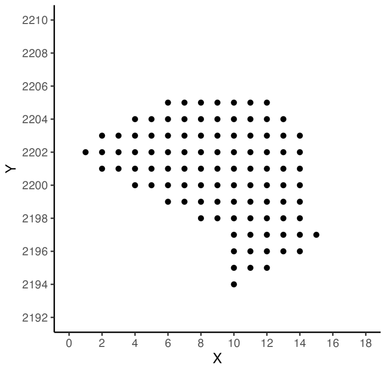

(a) Cell shape

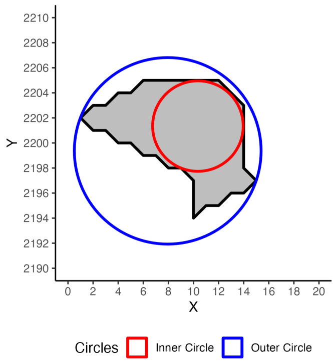

(b) Inscribing and circumscribing circles

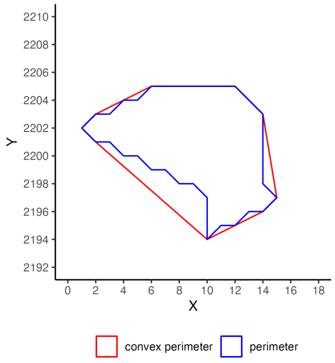

(c) Perimeter and convex perimeter

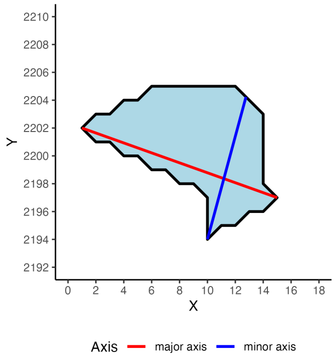

(d) Major and minor axis

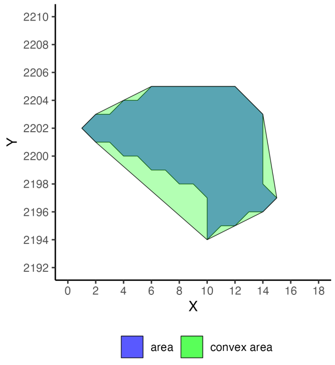

(e) Area and convex area

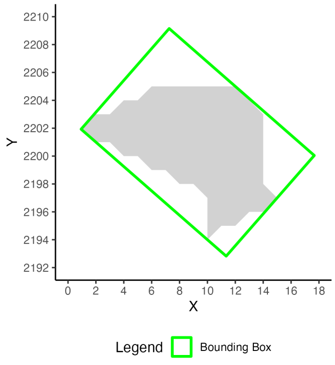

(f) Bounding box

Supplementary Figure 27: Illustrations of variables in cell metrics including (a) cell shape, (b) inscribing and circumscribing circles, (c) perimeter and convex perimeter, (d) major and minor axis, (e) area and convex area, and (f) bounding box.
