# Supplementary material for: BIDCell: Biologically-informed self-supervised learning for segmentation of subcellular spatial transcriptomics data
Source: Nat Commun. 2024 Jan 13;15:509. doi: 10.1038/s41467-023-44560-w (PMC10787788; doi:10.1038/s41467-023-44560-w)
Supplement: Supplementary file 3 — Reporting Summary [file 41467_2023_44560_MOESM3_ESM.pdf]

nature portfolio

Corresponding author(s): Jean Yang
Last updated by author(s): Dec 8, 2023

Reporting Summary

Nature Portfolio wishes to improve the reproducibility of the work that we publish. This form provides structure for consistency and transparency in reporting. For further information on Nature Portfolio policies, see our Editorial Policies and the Editorial Policy Checklist.

Statistics

For all statistical analyses, confirm that the following items are present in the figure legend, table legend, main text, or Methods section.

n/a	Confirmed	
☐	☒	The exact sample size (*n*) for each experimental group/condition, given as a discrete number and unit of measurement
☒	☐	A statement on whether measurements were taken from distinct samples or whether the same sample was measured repeatedly
☒	☐	The statistical test(s) used AND whether they are one- or two-sided<br>*Only common tests should be described solely by name; describe more complex techniques in the Methods section.*
☒	☐	A description of all covariates tested
☒	☐	A description of any assumptions or corrections, such as tests of normality and adjustment for multiple comparisons
☐	☒	A full description of the statistical parameters including central tendency (e.g. means) or other basic estimates (e.g. regression coefficient) AND variation (e.g. standard deviation) or associated estimates of uncertainty (e.g. confidence intervals)
☒	☐	For null hypothesis testing, the test statistic (e.g. *F*, *t*, *r*) with confidence intervals, effect sizes, degrees of freedom and *P* value noted<br>*Give P values as exact values whenever suitable.*
☒	☐	For Bayesian analysis, information on the choice of priors and Markov chain Monte Carlo settings
☒	☐	For hierarchical and complex designs, identification of the appropriate level for tests and full reporting of outcomes
☒	☐	Estimates of effect sizes (e.g. Cohen's *d*, Pearson's *r*), indicating how they were calculated

Our web collection on statistics for biologists contains articles on many of the points above.

Software and code

Policy information about availability of computer code

Data collection	No software was required/used to download the data.
Data analysis	BIDCell code is available at https://github.com/SydneyBioX/BIDCell, including code for data pre-processing, model training, inference, and extracting gene expression from segmented cells. CellSPA code is available at https://github.com/SydneyBioX/CellSPA. Version 0.1.0 was used for both BIDCell and CellSPA.<br>Information for other methods are as follows: Baysor v0.5.2 (https://github.com/kharchenkolab/Baysor); JSTA (https://github.com/wollmanlab/JSTA; version ccce064); Cellpose v2.1.1 (https://github.com/MouseLand/cellpose); Voronoi via SciPy v1.9.3; Watershed via OpenCV v4.6.0

For manuscripts utilizing custom algorithms or software that are central to the research but not yet described in published literature, software must be made available to editors and reviewers. We strongly encourage code deposition in a community repository (e.g. GitHub). See the Nature Portfolio guidelines for submitting code & software for further information.

Data

Policy information about availability of data

All manuscripts must include a data availability statement. This statement should provide the following information, where applicable:

- Accession codes, unique identifiers, or web links for publicly available datasets
- A description of any restrictions on data availability
- For clinical datasets or third party data, please ensure that the statement adheres to our policy

All datasets used in this study are publicly available and were downloaded from the following links (more details including accession codes are provided in Table 1). 10x Genomics Xenium breast cancer replicates 1 and 2: https://www.10xgenomics.com/products/xenium-in-situ/preview-dataset-human-breast. 10x Genomics Xenium mouse brain: https://www.10xgenomics.com/resources/datasets/fresh-frozen-mouse-brain-replicates-1-standard. NanoString CosMx NSCLC: https://nanostring.com/products/cosmx-spatial-molecular-imager/nsclc-ffpe-dataset/. Vizgen MERSCOPE melanoma2: https://info.vizgen.com/merscope-ffpe-solution (requiresfillingintheformtoaccess). The Stereo-seq E12.5 E1S3 data were downloaded from https://db.cngb.org/stomics/mosta/download/. Tumor Immune Single Cell Hub 2 (TISCH2) BRCA: http://tisch.comp-genomics.org/gallery/?cancer=BRCA&species=Human. 10x Chromium breast cancer: https://www.10xgenomics.com/products/xenium-in-situ/preview-dataset-human-breast. Allen Brain Map Mouse Whole Cortex and Hippocampus SMART-seq: https://portal.brain-map.org/atlases-and-data/rnaseq/mouse-whole-cortex-and-hippocampus-smart-seq. Human Lung Cell Atlas: https://beta.fastgenomics.org/p/hlca. TISCH-NSCLC: http://tisch.comp-genomics.org/gallery/?cancer=NSCLC&species=Human. TISCH-SKCM: http://tisch.comp-genomics.org/gallery/?cancer=SKCM&species=Human. The mouse embryo reference was downloaded from GEO database under accession code GSE119945 [https://www.ncbi.nlm.nih.gov/geo/query/acc.cgi?acc=GSE119945]. The TISCH-BRCA datasets were downloaded from GEO database under accession codes GSE110686 [https://www.ncbi.nlm.nih.gov/geo/query/acc.cgi?acc=GSE110686], GSE114727 [https://www.ncbi.nlm.nih.gov/geo/query/acc.cgi?acc=GSE114727], GSE138536 [https://www.ncbi.nlm.nih.gov/geo/query/acc.cgi?acc=GSE138536], GSE143423 [https://www.ncbi.nlm.nih.gov/geo/query/acc.cgi?acc=GSE143423], GSE176078 [https://www.ncbi.nlm.nih.gov/geo/query/acc.cgi?acc=GSE176078], GSE148673 [https://www.ncbi.nlm.nih.gov/geo/query/acc.cgi?acc=GSE148673], GSE150660 [https://www.ncbi.nlm.nih.gov/geo/query/acc.cgi?acc=GSE150660]; from EBI database under accession code E-MTAB-8107 [https://www.ebi.ac.uk/biostudies/arrayexpress/studies/E-MTAB-8107]; and from SRA under accession code SRP114962 [https://trace.ncbi.nlm.nih.gov/Traces/?view=study&acc=SRP114962]. The original published datasets of HLCA can be accessed under GEO accession number GSE135893 [https://www.ncbi.nlm.nih.gov/geo/query/acc.cgi?acc=GSE135893] for Banovich Kropski 2020; URL [https://www.synapse.org/#!Synapse:syn21041850] for Krasnow 2020; GSE128033 [https://www.ncbi.nlm.nih.gov/geo/query/acc.cgi?acc=GSE128033] for Lafyatis Rojas 2019; URL [https://explore.data.humancellatlas.org/projects/c4077b3c-5c98-4d26-a614-246d12c2e5d7] for Meyer 2019; GSE158127 [https://www.ncbi.nlm.nih.gov/geo/query/acc.cgi?acc=GSE158127] for Misharin 2021; GSE122960 [https://www.ncbi.nlm.nih.gov/geo/query/acc.cgi?acc=GSE122960] and GSE121611 [https://www.ncbi.nlm.nih.gov/geo/query/acc.cgi?acc=GSE121611] for Misharin Budinger 2018; European Genome-phenome Archive study ID EGAD00001005065 [https://ega-archive.org/datasets/EGAD00001005065] for Teichmann Meyer 2019. The TISCH-NSCLC datasets were downloaded from GEO database under accession codes GSE117570 [https://www.ncbi.nlm.nih.gov/geo/query/acc.cgi?acc=GSE117570], GSE127465 [https://www.ncbi.nlm.nih.gov/geo/query/acc.cgi?acc=GSE127465], GSE143423 [https://www.ncbi.nlm.nih.gov/geo/query/acc.cgi?acc=GSE143423], GSE148071 [https://www.ncbi.nlm.nih.gov/geo/query/acc.cgi?acc=GSE148071], GSE150660 [https://www.ncbi.nlm.nih.gov/geo/query/acc.cgi?acc=GSE150660]; and from EBI database under accession code E-MTAB-6149 [https://www.ebi.ac.uk/biostudies/arrayexpress/studies/E-MTAB-6149]. The SKCM datasets were downloaded from GEO database under accession codes GSE115978 [https://www.ncbi.nlm.nih.gov/geo/query/acc.cgi?acc=GSE115978], GSE120575 [https://www.ncbi.nlm.nih.gov/geo/query/acc.cgi?acc=GSE120575], GSE123139 [https://www.ncbi.nlm.nih.gov/geo/query/acc.cgi?acc=GSE123139], GSE139249 [https://www.ncbi.nlm.nih.gov/geo/query/acc.cgi?acc=GSE139249], GSE148190 [https://www.ncbi.nlm.nih.gov/geo/query/acc.cgi?acc=GSE148190], GSE72056 [https://www.ncbi.nlm.nih.gov/geo/query/acc.cgi?acc=GSE72056], GSE134388 [https://www.ncbi.nlm.nih.gov/geo/query/acc.cgi?acc=GSE134388], GSE159251 [https://www.ncbi.nlm.nih.gov/geo/query/acc.cgi?acc=GSE159251], GSE166181 [https://www.ncbi.nlm.nih.gov/geo/query/acc.cgi?acc=GSE166181], and GSE179373 [https://www.ncbi.nlm.nih.gov/geo/query/acc.cgi?acc=GSE179373]. Source data are provided with this paper.

Research involving human participants, their data, or biological material

Policy information about studies with human participants or human data. See also policy information about sex, gender (identity/presentation), and sexual orientation and race, ethnicity and racism.

Reporting on sex and gender	N/A
Reporting on race, ethnicity, or other socially relevant groupings	N/A
Population characteristics	N/A
Recruitment	N/A
Ethics oversight	N/A

Note that full information on the approval of the study protocol must also be provided in the manuscript.

Field-specific reporting

Please select the one below that is the best fit for your research. If you are not sure, read the appropriate sections before making your selection.

☒ Life sciences ☐ Behavioural & social sciences ☐ Ecological, evolutionary & environmental sciences

For a reference copy of the document with all sections, see nature.com/documents/nr-reporting-summary-flat.pdf

Life sciences study design

All studies must disclose on these points even when the disclosure is negative.

Sample size	We validated BIDCell on 6 publicly available subcellular spatial transcriptomics datasets. The datasets were captured using different leading commercial platforms (10x Genomics Xenium, NanoString CosMx, Vizgen MERSCOPE, and BGI Stereo-seq), on diverse tissue types (breast cancer, mouse brain, NSCLC, melanoma, and mouse embryo), and involved various numbers of genes (248, 313, 500, 960, and more). No statistical method was used to predetermine sample size, all data were used in our study. The datasets contained sufficient numbers of cells (around 100,000 each) for analysis.
Data exclusions	No data was excluded from this study.
Replication	We demonstrated the replicability of BIDCell using two replicated studies of breast cancer datasets captured using 10x Genomics Xenium. Cells segmented using BIDCell for the two replicates were highly correlated, and the tSNE plots showed a well-mixed population of cells between the two replicated studies. Furthermore, we validated BIDCell on 5 publicly available subcellular spatial transcriptomics datasets.
Randomization	N/A. We didn't have multiple experimental groups across biological samples.
Blinding	N/A. We did not have multiple experimental groups or treat the samples in a way to induce a measurable effect.

Reporting for specific materials, systems and methods

We require information from authors about some types of materials, experimental systems and methods used in many studies. Here, indicate whether each material, system or method listed is relevant to your study. If you are not sure if a list item applies to your research, read the appropriate section before selecting a response.

Materials & experimental systems

n/a	Involved in the study	
☒	☐	Antibodies
☒	☐	Eukaryotic cell lines
☒	☐	Palaeontology and archaeology
☒	☐	Animals and other organisms
☒	☐	Clinical data
☒	☐	Dual use research of concern
☒	☐	Plants

Methods

n/a	Involved in the study	
☒	☐	ChIP-seq
☒	☐	Flow cytometry
☒	☐	MRI-based neuroimaging
